# Molecular Control of Innate Immune Response to *Pseudomonas aeruginosa* Infection by Intestinal *let-7* in *Caenorhabditis elegans*

**DOI:** 10.1371/journal.ppat.1006152

**Published:** 2017-01-17

**Authors:** Lingtong Zhi, Yonglin Yu, Xueying Li, Daoyong Wang, Dayong Wang

**Affiliations:** Key Laboratory of Developmental Genes and Human Diseases in Ministry of Education, Medical School, Southeast University, Nanjing, China; Stanford University, UNITED STATES

## Abstract

The microRNA (miRNA) *let-7* is an important miRNA identified in *Caenorhabditis elegans* and has been shown to be involved in the control of innate immunity. The underlying molecular mechanisms for *let-7* regulation of innate immunity remain largely unclear. In this study, we investigated the molecular basis for intestinal *let-7* in the regulation of innate immunity. Infection with *Pseudomonas aeruginosa* PA14 decreased *let-7*::*GFP* expression. Intestine- or neuron-specific activity of *let-7* was required for its function in the regulation of innate immunity. During the control of innate immune response to *P*. *aeruginosa* PA14 infection, SDZ-24 was identified as a direct target for intestinal *let-7*. SDZ-24 was found to be predominantly expressed in the intestine, and *P*. *aeruginosa* PA14 infection increased SDZ-24::GFP expression. Intestinal *let-7* regulated innate immune response to *P*. *aeruginosa* PA14 infection by suppressing both the expression and the function of SDZ-24. Knockout or RNA interference knockdown of *sdz-24* dampened the resistance of *let-7* mutant to *P*. *aeruginosa* PA14 infection. Intestinal overexpression of *sdz-24* lacking 3’-UTR inhibited the susceptibility of nematodes overexpressing intestinal *let-7* to *P*. *aeruginosa* PA14 infection. In contrast, we could observed the effects of intestinal *let-7* on innate immunity in *P*. *aeruginosa* PA14 infected transgenic strain overexpressing *sdz-24* containing 3’-UTR. In the intestine, certain SDZ-24-mediated signaling cascades were formed for nematodes against the *P*. *aeruginosa* PA14 infection. Our results highlight the crucial role of intestinal miRNAs in the regulation of the innate immune response to pathogenic infection.

## Author Summary

Some microRNAs (miRNAs) have been identified recently to play important roles in the regulation of innate immunity in *Caenorhabditis elegans*. *let-7* is one of important miRNAs identified to be involved in the control of innate immune response. However, the underlying molecular mechanism for *let-7* in the regulation of innate immune response is still largely unknown. In *C*. *elegans*, *let-7* could function in both the intestine and the neurons to regulate the innate immunity. We here focused on the examination of molecular basis for the intestinal *let-7* in the regulation of innate immune response to *Pseudomonas aeruginosa* PA14 infection. We identified SDZ-24, an ortholog of human replication protein A1, as a direct target for intestinal *let-7* in the regulation of innate immune response. For the molecular mechanisms of intestinal *let-7* in the regulation of innate immunity, *let-7* might negatively regulate the function of SKN-1 by suppressing the expression and function of its target of SDZ-24. Our results imply the important function of intestinal miRNAs, such as *let-7*, in the regulation of innate immune response to pathogenic infection.

## Introduction

The free-living nematode *Caenorhabditis elegans* lives in soil, and potentially composts rich in the microorganisms, including those that are human microbial pathogens [1–2]. In the laboratory, once pathogenic bacteria can be deposited in the gut, they will invade the host cells, and even kill the nematodes through infectious processes. *C*. *elegans* usually responds to pathogen exposure by avoiding pathogens or activating an inducible innate immune system [3]. *C*. *elegans* has been considered as a useful model for the study of innate immunity at least based on the identification of virulence-related microbial genes and immune-based host genes [4]. Moreover, studies in *C*. *elegans* may potentially provide evolutionary and mechanistic insights into the signal transduction and the physiology of innate immunity [3]. Both genetic and functional genomic approaches in *C*. *elegans* have identified some conserved and important signaling pathways required for the control of innate immunity. Some of these identified conserved signal pathways involve p38 mitogen-activated protein kinase (MAPK), insulin, and TGF-β signaling pathways [5–8].

microRNAs (miRNAs) are a class of evolutionarily conserved non-coding RNAs with 19–22 nucleotides, and function to negatively regulate the gene expression [9]. miRNAs are encoded within the genome, and mature miRNAs post-transcriptionally regulate the gene expression by imperfectly binding to multiple target mRNAs [10]. miRNAs have been shown to be involved in the control of diverse fundamental biological processes, such as development, cell differentiation, apoptosis, and immune response [9, 11–13]. *C*. *elegans* is a powerful *in vivo* model to study how miRNAs regulate the gene expression and regulate various biological processes during the development [11, 14]. Recently, some miRNAs such as *mir-84*, *mir-241*, *mir-233*, *mir-251*, *mir-252*, and *mir-360* have been shown to be involved in the control of innate immunity in *C*. *elegans* [15–17].

*let-7* is one of the founding members of miRNA family firstly identified in *C*. *elegans via* forward genetic screen [18]. Some studies have suggested that *let-7* can act as a developmental switch to control the transition from larval to adult [18–19]. More recently, it has been shown that *let-7* was also involved in the control of innate immunity [20]. However, the underlying molecular mechanism for *let-7* in the regulation of innate immunity is still largely unclear. In the present study, we aimed to examine the molecular basis for intestinal *let-7* in the regulation of innate immunity. We identified SDZ-24 as a potential target of intestinal *let-7* in the regulation of innate immunity. In *C*. *elegans*, *sdz-24* gene encodes an ortholog of human replication protein A1 (RPA1) [21]. SKN-1, a homolog to Nrf transcription factors, plays an important role in pathogen resistance [22]. Our results suggest that intestinal *let-7* could directly target the SDZ-24, and regulated the innate immune response to *Pseudomonas aeruginosa* PA14 infection by suppressing the function of SDZ-24-SKN-1 signaling cascade. Our findings will not only aid our understanding the molecular basis for intestinal *let-7* in the regulation of innate immunity, but will also be helpful for further understanding the complex biological functions of *let-7* in animals.

## Results

### Effects of *P*. *aeruginosa* PA14 infection on *let-7* expression

**Fig 1:**
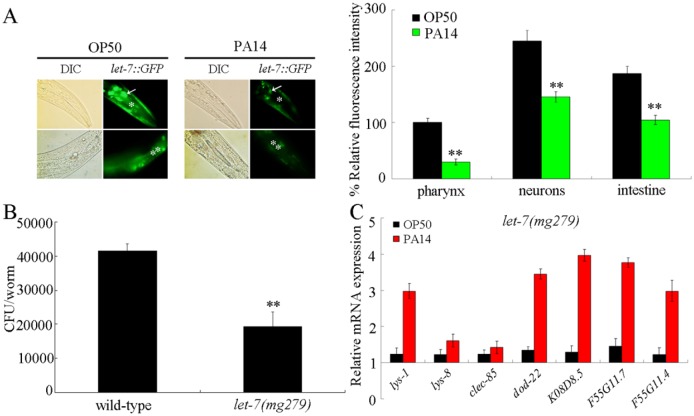
Response of *let-7* to *P*. *aeruginosa* PA14 infection. (A) Effect of *P*. *aeruginosa* PA14 infection on *let-7*::*GFP* expression. Arrowheads indicate the neurons. Pharynx (*) and intestine (**) were also indicated. Nematodes were infected with *P*. *aeruginosa* PA14 for 24-h. Thirty animals were examined. Bars represent mean ± SD. **P < 0.01 *vs* OP50. (B) Comparison of *P*. *aeruginosa* PA14 CFU between wild-type N2 and *let-7(mg279)* mutants infected with *P*. *aeruginosa* PA14. Bars represent mean ± SD. **P < 0.01 *vs* wild-type. (C) Quantitative real-time PCR analysis of expression patterns of the anti-microbial peptide genes in *let-7(mg279)* mutant infected with *P*. *aeruginosa* PA14. Normalized expression is presented relative to wild-type expression. Bars represent mean ± SD.

In *C*. *elegans*, *let-7* is expressed in almost all tissues except the germline [23]. Using the transgenic strain of *zaEx5*[*let-7*::*GFP*], we observed that exposure to *P*. *aeruginosa* PA14 at least significantly decreased the expression of *let-7*::*GFP* in the pharynx, the neurons, and the intestine of infected transgenic strain compared with exposure to *Escherichia coli* OP50 (Fig 1A). Therefore, *P*. *aeruginosa* PA14 infection may suppress the *let-7* expression in the body of nematodes.

### Mutation of *let-7* decreased the colony formation of *P*. *aeruginosa* PA14 and enhanced the innate immune response

A previous study has demonstrated that the loss-of-function mutant of *let-7(mg279)* was resistant to *P*. *aeruginosa* PA14 infection as indicated by the increased survival compared with that in infected wild-type nematodes [20]. Meanwhile, we found that the loss-of-function mutation of *let-7* significantly decreased the colony-forming unit (CFU) of *P*. *aeruginosa* PA14 in the body of nematodes (Fig 1B). The normal accumulation of PA14::GFP in the lumen of pharynx of *let-7(mg279)* mutant implies that mutation of *let-7* did not cause the deficit in the feeding of *P*. *aeruginosa* PA14 (S1 Fig). Moreover, we investigated the effect of loss-of-function mutation of *let-7* on the expression of antimicrobial genes. The examined antimicrobial genes were *lys-1*, *lys-8*, *clec-85*, *dod-22*, *K08D8*.*5*, *F55G11*.*7*, and *F55G11*.*4*. *P*. *aeruginosa* PA14 could induce the significant increase in transcriptional expression of these antimicrobial genes in *C*. *elegans* [24]. We observed the noticeable increase in transcriptional expression of some antimicrobial genes (*lys-1*, *dod-22*, *K08D8*.*5*, *F55G11*.*7*, and *F55G11*.*4*) in *P*. *aeruginosa* PA14 infected *let-7(mg279)* mutant compared with *P*. *aeruginosa* PA14 infected wild-type nematodes (Fig 1C). On plates fed with *E*. *coli* OP50, the expression levels of these antimicrobial genes in *let-7(mg279)* mutant were similar to those in wild-type nematodes (Fig 1C). The *let-7(mg279)* mutant showed the similar brood size to wild-type (S2 Fig), suggesting that the observed resistance to PA14 infection in *let-7(mg279)* mutant is not the potential effect of *let-7* mutation on fecundity. These results suggest that the loss-of-function mutation of *let-7* may suppress the *P*. *aeruginosa* PA14 colonization in the body of nematodes, and induce an elevated immune competence.

### Tissue-specific activity of *let-7* in the regulation of innate immune response to *P*. *aeruginosa* PA14 infection

**Fig 2:**
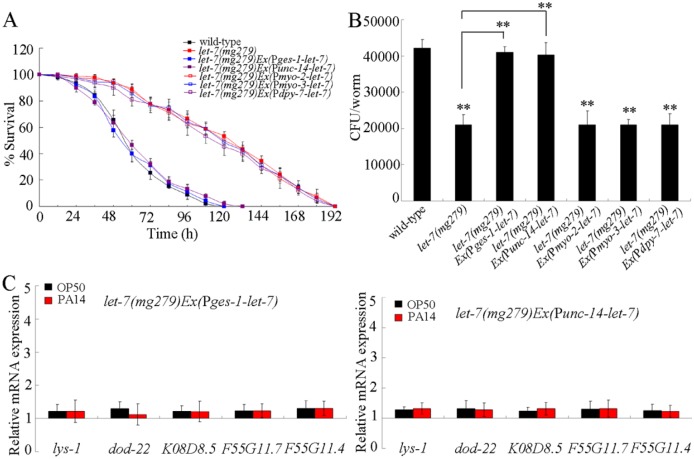
Tissue-specific activity of *let-7* in the regulation of innate immunity. (A) Tissue-specific activity of *let-7* in the regulation of survival in *P*. *aeruginosa* PA14 infected nematodes. Statistical comparisons of the survival plots indicate that, after *P*. *aeruginosa* PA14 infection, the survival of *let-7(mg279)Ex(*P*ges-1-let-7)* (*P* < 0.0001) or *let-7(mg279)Ex(*P*unc-14-let-7)* (*P* < 0.0001) was significantly different from that of *let-7(mg279)*, and the survival of *let-7(mg279)Ex(*P*myo-2-let-7)* (*P* = 0.9643), *let-7(mg279)Ex(*P*myo-3-let-7)* (*P* = 0.9234), or *let-7(mg279)Ex(*P*dpy-7-let-7)* (*P* = 0.9545) was not significantly different from that of *let-7(mg279)*. (B) Tissue-specific activity of *let-7* in the regulation of *P*. *aeruginosa* PA14 CFU in the body of nematodes. (C) Tissue-specific activity of *let-7* in the regulation of expression patterns of anti-microbial genes in *P*. *aeruginosa* PA14 infected nematodes. Normalized expression is presented relative to wild-type expression. Bars represent mean ± SD. **P < 0.01 *vs* wild-type (if not specially indicated).

In this study, we next investigated the intestine-, neuron-, pharynx-, muscle-, or hypodermis-specific activity of *let-7* in the regulation of innate immune response to *P*. *aeruginosa* PA14 infection. After *P*. *aeruginosa* PA14 infection, expression of *let-7* in the pharynx, the muscle, or the hypodermis did not significantly affect the survival and the CFU of *P*. *aeruginosa* PA14 in *let-7(mg279)* mutant (Fig 2A and 2B). In contrast, after *P*. *aeruginosa* PA14 infection, expression of *let-7* in the intestine or the neurons significantly reduced the survival, increased the CFU of *P*. *aeruginosa* PA14, and decreased the expressions of antimicrobial genes (*lys-1*, *dod-22*, *K08D8*.*5*, *F55G11*.*7*, and *F55G11*.*4*) in *let-7(mg279)* mutant (Fig 2). Additionally, the survival, the CFU of *P*. *aeruginosa* PA14, or the expression patterns of antimicrobial genes (*lys-1*, *dod-22*, *K08D8*.*5*, *F55G11*.*7*, and *F55G11*.*4*) in *P*. *aeruginosa* PA14 infected transgenic strain of *let-7(mg279)Ex(*P*ges-1-let-7)* or *let-7(mg279)Ex(*P*unc-14-let-7)* were similar to those in *P*. *aeruginosa* PA14 infected wild-type nematodes (Fig 2B). Therefore, *let-7* may act in the intestine or the neurons to regulate the innate immune response to *P*. *aeruginosa* PA14 infection.

### Nematodes overexpressing *let-7* in the intestine or the neurons were susceptible to *P*. *aeruginosa* PA14 infection

To confirm the function of *let-7* in the regulation of innate immunity, we investigated the effects of *let-7* overexpression in the intestine or the neurons on innate immune response to *P*. *aeruginosa* PA14 infection. After exposure to *P*. *aeruginosa* PA14, nematodes overexpressing *let-7* in the intestine or the neurons exhibited the significant decrease in survival compared with wild-type nematodes (S3A Fig). After exposure to *P*. *aeruginosa* PA14, overexpression of *let-7* in the intestine or the neurons also resulted in a significant increase in CFU of *P*. *aeruginosa* PA14 in the body compared with wild-type nematodes (S3B Fig). Moreover, after exposure to *P*. *aeruginosa* PA14, nematodes overexpressing *let-7* in the intestine or the neurons showed the decreased expression in antimicrobial genes (*lys-1*, *dod-22*, *K08D8*.*5*, *F55G11*.*7*, and *F55G11*.*4*) compared with wild-type nematodes (S3C Fig). These results suggest that the overexpression of *let-7* in the intestine or the neurons may induce a susceptible property of nematodes to *P*. *aeruginosa* PA14 infection.

### Identification of candidate *let-7* targets involved in the control of innate immune response to *P*. *aeruginosa* PA14 infection

**Fig 3:**
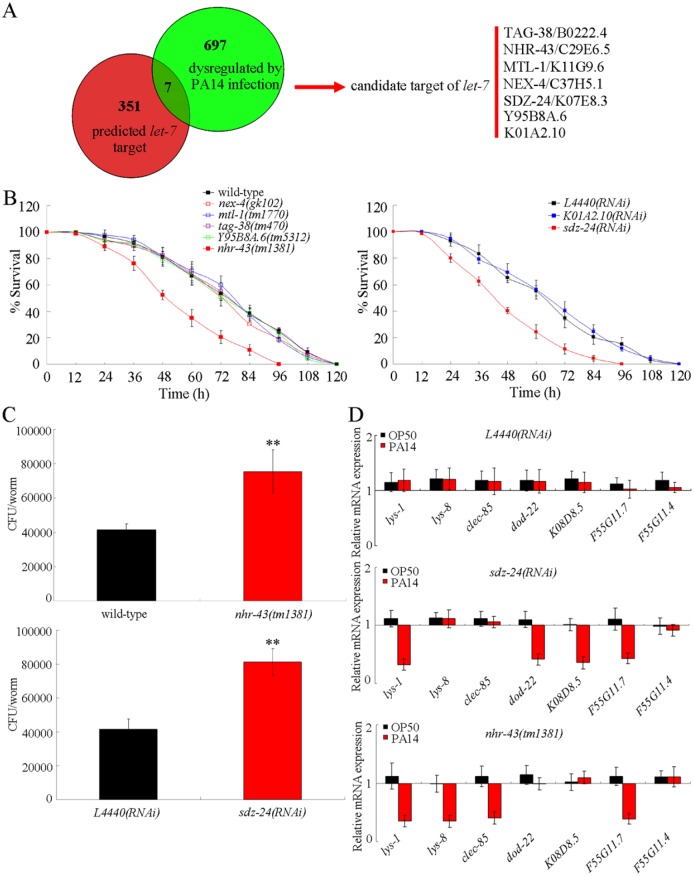
Identification of candidate targets for *let-7* in the regulation of innate immune response to *P*. *aeruginosa* PA14 infection. (A) Search for the candidate targets for *let-7* in the regulation of innate immune response to *P*. *aeruginosa* PA14 infection. (B) Effects of mutation or RNAi knockdown of candidate targeted genes of *let-7* on survival in *P*. *aeruginosa* PA14 infected nematodes. Statistical comparisons of the survival plots indicate that, after *P*. *aeruginosa* PA14 infection, the survival of *nhr-43(tm1381)* mutant was significantly different from that of wild-type (*P* < 0.0001), and the survival of *sdz-24(RNAi)* was significantly different from that of *L4440(RNAi)* nematodes (*P* < 0.0001). (C) Effects of mutation or RNAi knockdown of candidate targeted genes of *let-7* on CFU of *P*. *aeruginosa* PA14 in the body of nematodes. (D) Effects of mutation or RNAi knockdown of candidate targeted genes of *let-7* on expression patterns of anti-microbial genes in *P*. *aeruginosa* PA14 infected nematodes. Normalized expression is presented relative to wild-type expression for *L4440(RNAi)* or *nhr-43(tm1381)*. Normalized expression is presented relative to *L4440(RNAi)* expression for *sdz-24(RNAi)*. *L4440(RNAi)*, empty vector RNAi. Bars represent mean ± SD. **P < 0.01 *vs* wild-type or *L4440(RNAi)*.

To identify the potential targets for *let-7* in the regulation of innate immune response to *P*. *aeruginosa* PA14 infection, we first used the TargetScan software to predict the potential targets for *let-7* by searching for the presence of conserved sites that match the seed region of *let-7*, and found 351 potential targets for *let-7* in *C*. *elegans* (Fig 3A). In *C*. *elegans*, a previous study has suggested that 697 genes could be dysregulated by *P*. *aeruginosa* PA14 infection [25]. Among the predicted 351 targeted genes, 7 genes (*tag-38*, *nhr-43*, *mtl-1*, *nex-4*, *sdz-24*, *Y95B8A*.*6*, and *K01A2*.*10*) were also dysregulated by *P*. *aeruginosa* PA14 infection (Fig 3A).

Using the corresponding mutants or RNA interference (RNAi) knockdown animals for these 7 genes, we found that only loss-of-mutation of *nhr-43* gene or RNAi knockdown of *sdz-24* gene significantly influenced the survival of infected nematodes after *P*. *aeruginosa* PA14 infection (Fig 3B). After *P*. *aeruginosa* PA14 infection, loss-of-mutation of *nhr-43* gene or RNAi knockdown of *sdz-24* gene significantly reduced the survival in nematodes (Fig 3B). In *C*. *elegans*, NHR-43 is a nuclear hormone receptor (NHR), and SDZ-24 is a SKN-1-dependent zygotic protein. Meanwhile, loss-of-mutation of *nhr-43* gene or RNAi knockdown of *sdz-24* gene significantly enhanced the colony formation of *P*. *aeruginosa* PA14 in the body of nematodes (Fig 3C). Moreover, loss-of-mutation of *nhr-43* gene caused the decreased expression in antimicrobial genes (*lys-1*, *lys-8*, *clec-85*, and *F55G11*.*7*), and RNAi knockdown of *sdz-24* gene resulted in the decreased expression in antimicrobial genes (*lys-1*, *dod-22*, *K08D8*.*5*, and *F55G11*.*7*) (Fig 3D).

### Genetic interaction between *let-7* and *nhr-43* or *sdz-24* in the regulation of innate immune response to *P*. *aeruginosa* PA14 infection

**Fig 4:**
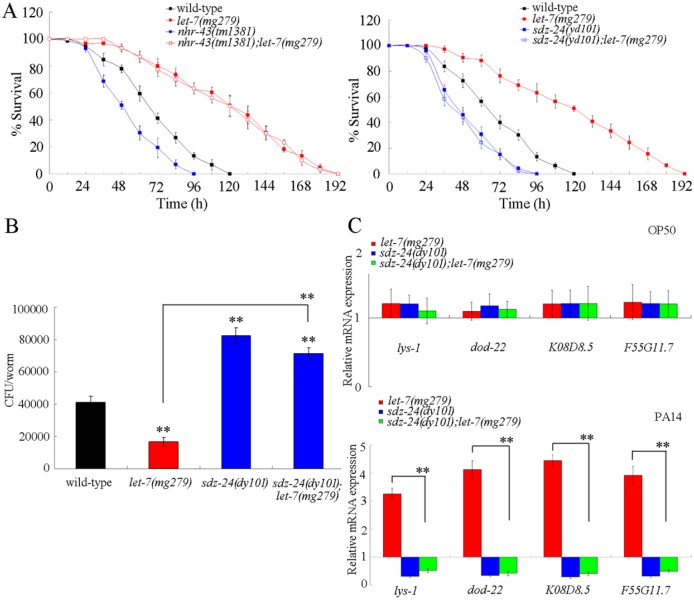
Genetic interaction between *let-7* and *sdz-24* in the regulation of innate immune response to *P*. *aeruginosa* PA14 infection. (A) Genetic interaction between *let-7* and *sdz-24* in the regulation of survival in *P*. *aeruginosa* PA14 infected nematodes. Statistical comparisons of the survival plots indicate that, after *P*. *aeruginosa* PA14 infection, the survival of *nhr-43(tm1381);let-7(mg279)* was not significantly different from that of *let-7(mg279)* (*P* = 0.9213), and the survival of *sdz-24(yd101);let-7(mg279)* was significantly different from that of *let-7(mg279)* (*P* < 0.0001). (B) Genetic interaction between *let-7* and *sdz-24* in the regulation of CFU of *P*. *aeruginosa* PA14 in the body of nematodes. (C) Genetic interaction between *let-7* and *sdz-24* in the regulation of expression patterns of anti-microbial genes in *P*. *aeruginosa* PA14 infected nematodes. Normalized expression is presented relative to wild-type expression. Bars represent mean ± SD. **P < 0.01 *vs* wild-type (if not specially indicated).

To confirm the role of *nhr-43* or *sdz-24* as the targeted gene of *let-7*, we constructed the double mutants of *nhr-43(tm1381);let-7(mg279)* and *sdz-24(yd101);let-7(mg279)*. We assumed that the *nhr-43* or *sdz-24* mutation would suppress the phenotype in nematodes with *let-7* mutation, if *nhr-43* or *sdz-24* is the targeted gene of *let-7* in nematodes. To confirmed the function of SDZ-24 in the regulation of innate immunity to *P*. *aeruginosa* PA14 infection, we generated the knockout strain for *sdz-24* (*sdz-24(yd101)*) using the clustered, regularly interspersed, short palindromic repeats (CRISPR) RNA-guided Cas9 technique [26]. The related deletion information was shown in S4 Fig. The survival, CFU of *P*. *aeruginosa* PA14, and expression patterns of antimicrobial genes in *P*. *aeruginosa* PA14 infected *sdz-24(yd101)* were similar to those in *P*. *aeruginosa* PA14 infected *sdz-24(RNAi)* nematodes (Fig 4). The *sdz-24(yd101)* mutant also had the normal accumulation of PA14::GFP in the lumen of pharynx, which was similar to that in wild-type nematodes (S1 Fig).

After *P*. *aeruginosa* PA14 infection, we found that mutation of *sdz-24* gene significantly reduced the survival in *let-7(mg279)* mutant; however, mutation of *nhr-43* gene did not obviously affect the survival in *let-7(mg279)* mutant (Fig 4A). These results suggest that the SDZ-24, not the NHR-43, is the direct target for *let-7* in the regulation of innate immune response to *P*. *aeruginosa* PA14 infection.

The assays on CFU of *P*. *aeruginosa* PA14 and expression pattern of antimicrobial genes further confirmed the role of SDZ-24 as the direct target for *let-7* in the regulation of innate immunity. Mutation of *sdz-24* gene significantly increased the CFU of *P*. *aeruginosa* PA14 in the body of *let-7(mg279)* mutant (Fig 4B). Moreover, mutation of *sdz-24* gene significantly decreased the expression of antimicrobial genes ((*lys-1*, *dod-22*, *K08D8*.*5*, and *F55G11*.*7*) in *let-7(mg279)* mutant (Fig 4C).

### Effects of *let-7* mutation on SDZ-24::GFP expression

In *C*. *elegans*, SDZ-24::GFP is primarily expressed in the posterior of intestine (S5 Fig). After *P*. *aeruginosa* PA14 infection, the expression of SDZ-24::GFP was significantly increased (S5 Fig), which is different from the effect of the *P*. *aeruginosa* PA14 infection on expression of *let-7*::*GFP* as indicated above.

Moreover, loss-of-function mutation of *let-7* significantly increased the SDZ-24::GFP expression under both the *E*. *coli* OP50 exposure condition and the *P*. *aeruginosa* PA14 exposure condition (S5 Fig), suggesting that *let-7* may potentially suppress the expression of SDZ-24.

### Intestinal RNAi of *sdz-24* gene caused a susceptible property of nematodes to *P*. *aeruginosa* PA14 infection

**Fig 5:**
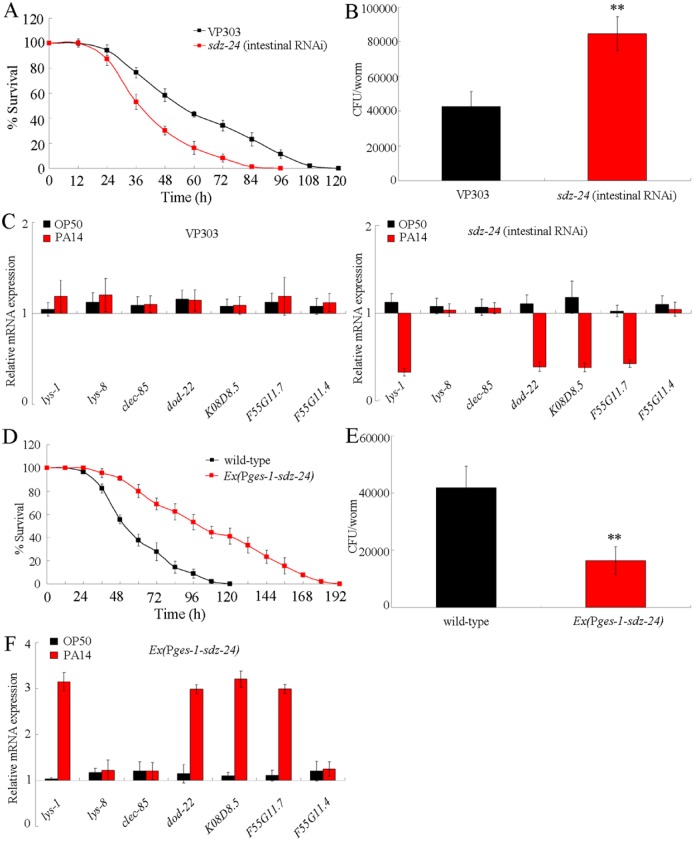
Function of SDZ-24 in the regulation of innate immune response to *P*. *aeruginosa* PA14 infection. (A) Effects of intestinal RNAi of *sdz-24* gene on survival in *P*. *aeruginosa* PA14 infected nematodes. Statistical comparison of the survival plots indicate that, after *P*. *aeruginosa* PA14 infection, the survival of nematodes with intestinal RNAi of *sdz-24* gene was significantly different from that of VP303 strain (*P* < 0.0001). Bars represent mean ± SD. (B) Effects of intestinal RNAi of *sdz-24* gene on CFU of *P*. *aeruginosa* PA14 in the body of nematodes. Bars represent mean ± SD. **P < 0.01 *vs* VP303. (C) Effects of intestinal RNAi of *sdz-24* gene on expression patterns of anti-microbial genes in *P*. *aeruginosa* PA14 infected nematodes. Normalized expression is presented relative to wild-type expression for VP303. Normalized expression is presented relative to VP303 for *sdz-24(intestinal RNAi)*. Bars represent mean ± SD. (D) Effects of *sdz-24* overexpression in the intestine on survival in *P*. *aeruginosa* PA14 infected nematodes. Statistical comparisons of the survival plots indicate that, after *P*. *aeruginosa* PA14 infection, the survival of nematodes with *sdz-24* overexpression in the intestine was significantly different from that of wild-type nematodes (*P* < 0.0001). Bars represent mean ± SD. (E) Effects of *sdz-24* overexpression in the intestine on CFU of *P*. *aeruginosa* PA14 in the body of nematodes. Bars represent mean ± SD. **P < 0.01 *vs* wild-type. (F) Effects of *sdz-24* overexpression in the intestine on expression patterns of anti-microbial genes in *P*. *aeruginosa* PA14 infected nematodes. Normalized expression is presented relative to wild-type expression. Bars represent mean ± SD.

Considering the fact that the *sdz-24* gene is pronounced expressed in the intestine, we investigated the effects of intestinal RNAi of *sdz-24* gene on innate immune response to *P*. *aeruginosa* PA14 infection using the VP303 strain as the intestinal RNAi tool [27]. After *P*. *aeruginosa* PA14 infection, intestinal RNAi of *sdz-24* gene significantly reduced the survival, enhanced the CFU of *P*. *aeruginosa* PA14 in the body of nematodes, and decreased the expression of antimicrobial genes (*lys-1*, *dod-22*, *K08D8*.*5*, and *F55G11*.*7*) (Fig 5A–5C). We also employed another intestinal RNAi tool of *sid-1(qt9)* to perform the RNAi assay [28]. We obtained the similar results (S6 Fig). Therefore, intestinal RNAi of *sdz-24* gene may induce a susceptible property of nematodes to *P*. *aeruginosa* PA14 infection.

### Effects of intestinal *sdz-24* overexpression on innate immune response to *P*. *aeruginosa* PA14 infection

Different from the effects of intestinal RNAi of *sdz-24* gene on innate immune response to *P*. *aeruginosa* PA14 infection, we observed that overexpression of *sdz-24* gene in the intestine led to significant increase in the survival, decrease in the CFU of *P*. *aeruginosa* PA14 in the body of nematodes, and enhancement of the expression of antimicrobial genes (*lys-1*, *dod-22*, *K08D8*.*5*, and *F55G11*.*7*) after *P*. *aeruginosa* PA14 infection (Fig 5D–5F). These results suggest that overexpression of *sdz-24* gene in the intestine may induce a resistance for nematodes against the toxic effects from *P*. *aeruginosa* PA14 infection, which further confirms the important function of SDZ-24 in the intestine in the regulation of innate immunity.

### Interaction between *let-7* and SDZ-24 in the intestine in the regulation of innate immunity to *P*. *aeruginosa* PA14 infection

**Fig 6:**
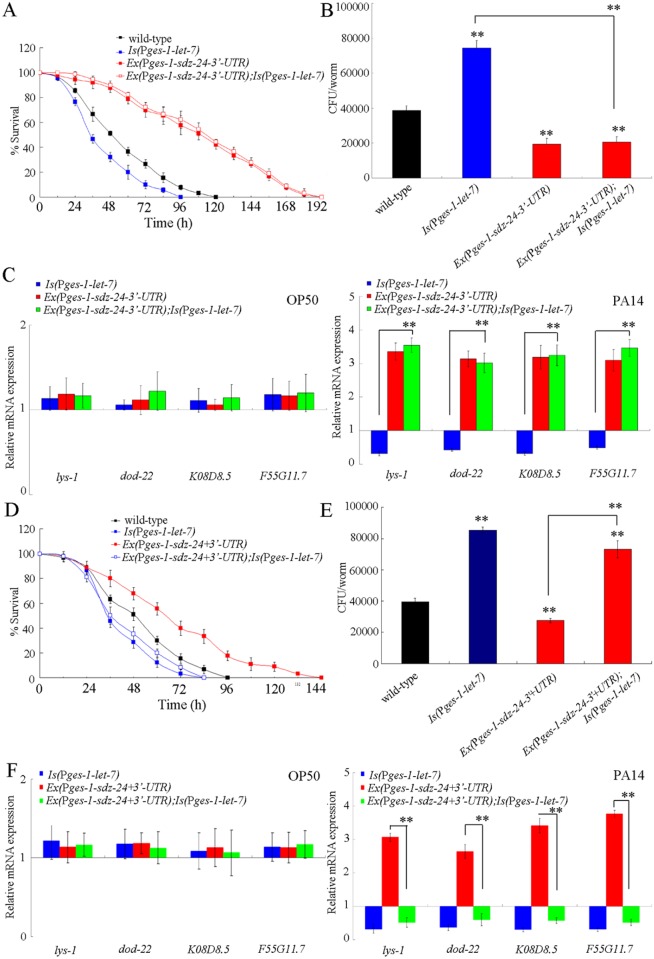
Effects of intestinal overexpression of *sdz-24* lacking 3’-UTR or containing 3’-UTR on innate immune response to *P*. *aeruginosa* PA14 infection in nematodes overexpressing intestinal *let-7*. (A) Effects of intestinal overexpression of *sdz-24* lacking 3’-UTR on survival in *P*. *aeruginosa* PA14 infected nematodes overexpressing intestinal *let-7*. Statistical comparisons of the survival plots indicate that, after *P*. *aeruginosa* PA14 infection, the survival of transgenic strain of *Ex(*P*ges-1-sdz-24-3’-UTR);Is(*P*ges-1-let-7)* was significantly different from that of transgenic strain of *Is(*P*ges-1-let-7)* (*P* < 0.0001). (B) Effects of intestinal overexpression of *sdz-24* lacking 3’-UTR on CFU of *P*. *aeruginosa* PA14 in *P*. *aeruginosa* PA14 infected nematodes overexpressing intestinal *let-7*. (C) Effects of intestinal overexpression of *sdz-24* lacking 3’-UTR on expression patterns of anti-microbial genes in *P*. *aeruginosa* PA14 infected nematodes overexpressing intestinal *let-7*. Normalized expression is presented relative to wild-type expression. (D) Effects of intestinal overexpression of *sdz-24* containing 3’-UTR on survival in *P*. *aeruginosa* PA14 infected nematodes overexpressing intestinal *let-7*. Statistical comparisons of the survival plots indicate that, after *P*. *aeruginosa* PA14 infection, the survival of transgenic strain of *Ex(*P*ges-1-sdz-24+3’-UTR);Is(*P*ges-1-let-7)* was significantly different from that of transgenic strain of *Ex(*P*ges-1-sdz-24+3’-UTR)* (*P* < 0.0001). (E) Effects of intestinal overexpression of *sdz-24* containing 3’-UTR on CFU of *P*. *aeruginosa* PA14 in *P*. *aeruginosa* PA14 infected nematodes overexpressing intestinal *let-7*. (F) Effects of intestinal overexpression of *sdz-24* containing 3’-UTR on expression patterns of anti-microbial genes in *P*. *aeruginosa* PA14 infected nematodes overexpressing intestinal *let-7*. Normalized expression is presented relative to wild-type expression. -, lacking 3’-UTR; +, containing 3’-UTR. Bars represent mean ± SD. **P < 0.01 *vs* wild-type (if not specially indicated).

To further determine the genetic interaction between *let-7* and SDZ-24 in the intestine in the regulation of innate immunity, we introduced the *sdz-24* lacking 3’-UTR driven by *ges-1* promoter, an intestine-specific promoter, into the nematodes overexpressing intestinal *let-7*. After *P*. *aeruginosa* PA14 infection, the transgenic strain of *Ex(*P*ges-1-sdz-24-3’-UTR);Is(*P*ges-1-let-7)* exhibited the similar survival, CFU of *P*. *aeruginosa* PA14, and expression patterns of antimicrobial genes (*lys-1*, *dod-22*, *K08D8*.*5*, and *F55G11*.*7*) to those in the transgenic strain of *Ex*(P*ges-1-sdz-24-3’-UTR)* (Fig 6A–6C). Meanwhile, the survival, CFU of *P*. *aeruginosa* PA14, and expression patterns of antimicrobial genes (*lys-1*, *dod-22*, *K08D8*.*5*, and *F55G11*.*7*) in *P*. *aeruginosa* PA14 infected transgenic strain of *Ex(*P*ges-1-sdz-24-3’-UTR);Is(*P*ges-1-let-7)* were significantly different from those in *P*. *aeruginosa* PA14 infected transgenic strain of *Is(*P*ges-1-let-7)* (Fig 6A–6C). These results suggest that the overexpression of *sdz-24* gene lacking 3’-UTR in the intestine may effectively suppress the susceptible property of nematodes overexpressing intestinal *let-7*. Very different from these, we found that intestinal overexpression of *let-7* could significantly reduce the survival, enhance the CFU of *P*. *aeruginosa* PA14, and decrease the expressions of antimicrobial genes (*lys-1*, *dod-22*, *K08D8*.*5*, and *F55G11*.*7*) in *P*. *aeruginosa* PA14 infected transgenic strain of *Ex(*P*ges-1-sdz-24+3’-UTR);Is(*P*ges-1-let-7)* (Fig 6D–6F), suggesting the effects of intestinal expression of *let-7* on innate immunity.

### *In vivo* 3’-UTR binding assay of *sdz-24*

**Fig 7:**
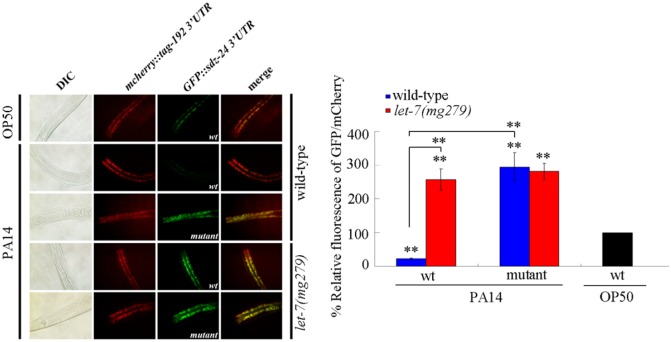
Fluorescence images of the *sdz-24*-3’-UTR GFP reporter in nematodes grown on *E*. *coli* OP50 or *P*. *aeruginosa* PA14. Bars represent mean ± SD. **P < 0.01 *vs* wt on OP50 (if not specially indicated).

To further confirm whether SDZ-24 is a direct target of *let-7*, we constructed a GFP vector driven by *ges-1* promoter, which contained the 3’-UTR of the *sdz-24* (P*ges-1*::*GFP-3’-UTR (sdz-24 wt)* or P*ges-1*::*GFP-3’-UTR (sdz-24 mut)*). Because *let-7* can not bind to the *tag-192* 3’-UTR, a P*ges-1*::*mCherry-3’-UTR (tag-192)* construct was used as an internal control. After *P*. *aeruginosa* PA14 infection, the GFP expression was noticeably reduced in wild-type nematodes; however, mutagenesis of the putative binding site for *let-7* in *sdz-24* 3’-UTR abolished this inhibition of GFP expression in wild-type nematodes (Fig 7). Moreover, after *P*. *aeruginosa* PA14 infection, the GFP expression was much higher in *let-7(mg279)* mutant than that in wild-type nematodes (Fig 7). These results further confirmed that *let-7* may suppress the function of SDZ-24 through binding to its 3’-UTR and inhibiting its translation in *P*. *aeruginosa* PA14 infected nematodes.

### Interaction between *sdz-24* and genes encoding some known signaling pathways in the regulation of innate immune response to *P*. *aeruginosa* PA14 infection

**Fig 8:**
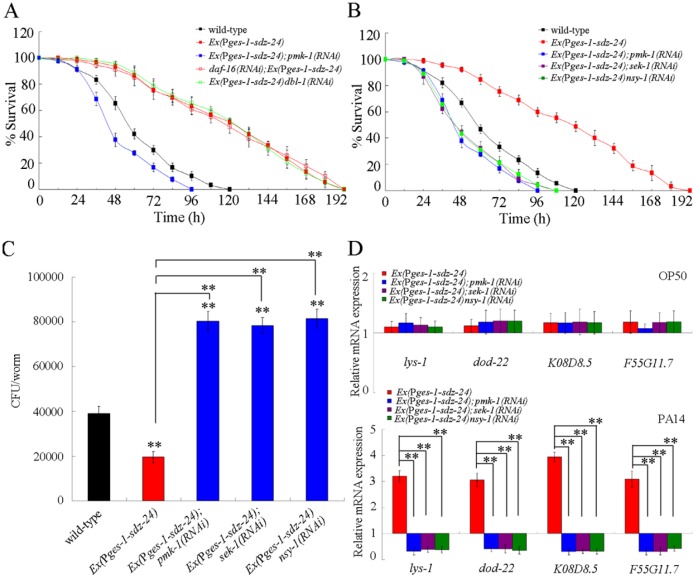
Genetic interactions between SDZ-24 and some known signaling pathways in the regulation of innate immune response to *P*. *aeruginosa* PA14 infection. (A) Genetic interaction between SDZ-24 and PMK-1, DAF-16, or DBL-1 in the regulation of survival in *P*. *aeruginosa* PA14 infected nematodes. Statistical comparisons of the survival plots indicate that, after *P*. *aeruginosa* PA14 infection, the survival of *Ex(*P*ges-1-sdz-24);pmk-1(RNAi)* (*P* < 0.0001) was significantly different from that of *Ex(*P*ges-1-sdz-24)*, and the survival of *daf-16(RNAi);Ex(*P*ges-1-sdz-24)* (*P* = 0.9463) or *Ex(*P*ges-1-sdz-24);dbl-1(RNAi)* (*P* = 0.9619) was not significantly different from that of *Ex(*P*ges-1-sdz-24)*. (B) Genetic interaction between SDZ-24 and PMK-1, SEK-1, or NSY-1 in the regulation of survival in *P*. *aeruginosa* PA14 infected nematodes. Statistical comparisons of the survival plots indicate that, after *P*. *aeruginosa* PA14 infection, the survival of *Ex(*P*ges-1-sdz-24);pmk-1(RNAi)*, *Ex(*P*ges-1-sdz-24);sek-1(RNAi)*, or *Ex(*P*ges-1-sdz-24);nsy-1(RNAi)* was significantly different from that of *Ex(*P*ges-1-sdz-24)* (*P* < 0.0001). (C) Genetic interaction between SDZ-24 and PMK-1, SEK-1, or NSY-1 in the regulation of CFU of *P*. *aeruginosa* PA14 in *P*. *aeruginosa* PA14 infected nematodes. (D) Genetic interaction between SDZ-24 and PMK-1, SEK-1, or NSY-1 in the regulation of expression patterns of anti-microbial genes in *P*. *aeruginosa* PA14 infected nematodes. Normalized expression is presented relative to wild-type expression. Bars represent mean ± SD. **P < 0.01 *vs* wild-type (if not specially indicated).

To determine the intestinal SDZ-24-mediated signaling pathway in the control of innate immunity, we investigated the genetic interaction between SDZ-24 and some known signaling pathways involved in the control of innate immune response to *P*. *aeruginosa* PA14 infection. In *C*. *elegans*, *daf-16* gene encodes a FOXO transcription factor in insulin signaling pathway, and *dbl-1* gene encodes a TGF-β ligand in TGF-β signaling pathway. In the p38 MAPK signaling pathway, *pmk-1* gene encoded a MAPK, *sek-1* gene encoded a MAPK kinase (MAPKK), and *nsy-1* gene encoded a MAPK kinase kinase (MAPKKK). After *P*. *aeruginosa* PA14 infection, RNAi knockdown of *daf-16* or *dbl-1* gene did not significantly affect the survival in transgenic nematodes overexpressing intestinal *sdz-24*; however, RNAi knockdown of *pmk-1*, *sek-1*, or *nsy-1* gene significantly suppressed the survival in transgenic nematodes overexpressing intestinal *sdz-24* (Fig 8A and 8B), suggesting that RNAi knockdown of genes encoding the p38 MAPK signaling pathway may potentially inhibit the resistant property of transgenic nematodes overexpressing intestinal *sdz-24* to *P*. *aeruginosa* PA14 infection. RNAi knockdown of *pmk-1*, *sek-1*, or *nsy-1* gene also significantly increased the CFU of *P*. *aeruginosa* PA14 in the body of *P*. *aeruginosa* PA14 infected transgenic nematodes overexpressing intestinal *sdz-24* (Fig 8C). Moreover, RNAi knockdown of *pmk-1*, *sek-1*, or *nsy-1* gene significantly decreased the expression of antimicrobial genes (*lys-1*, *dod-22*, *K08D8*.*5*, and *F55G11*.*7*) in *P*. *aeruginosa* PA14 infected transgenic nematodes overexpressing intestinal *sdz-24* (Fig 8D).

To confirm the interaction between SDZ-24 and p38 MAPK signaling pathway in the regulation of innate immunity to *P*. *aeruginosa* PA14 infection, we further examined the effect of *sdz-24* mutation on expression of some immune effectors (*T24B8*.*5*, *F08G5*.*6*, and *F35E12*.*5*) for the p38 MAPK signaling pathway [29–30]. However, we found that mutation of *sdz-24* did not significantly affect the expression of these immune effectors for the p38 MAPK signaling pathway (S7A Fig). Moreover, mutation of *sdz-24* also did not significantly affect the expression of phosphorylated PMK-1 compared with wild-type (S7B Fig). Therefore, SDZ-24 may actually do not act upstream of the p38 MAPK signaling pathway to regulate innate immunity in *P*. *aeruginosa* PA14 infected nematodes.

### Genetic interaction of *sdz-24* with *skn-1* in the regulation of innate immune response to *P*. *aeruginosa* PA14 infection

**Fig 9:**
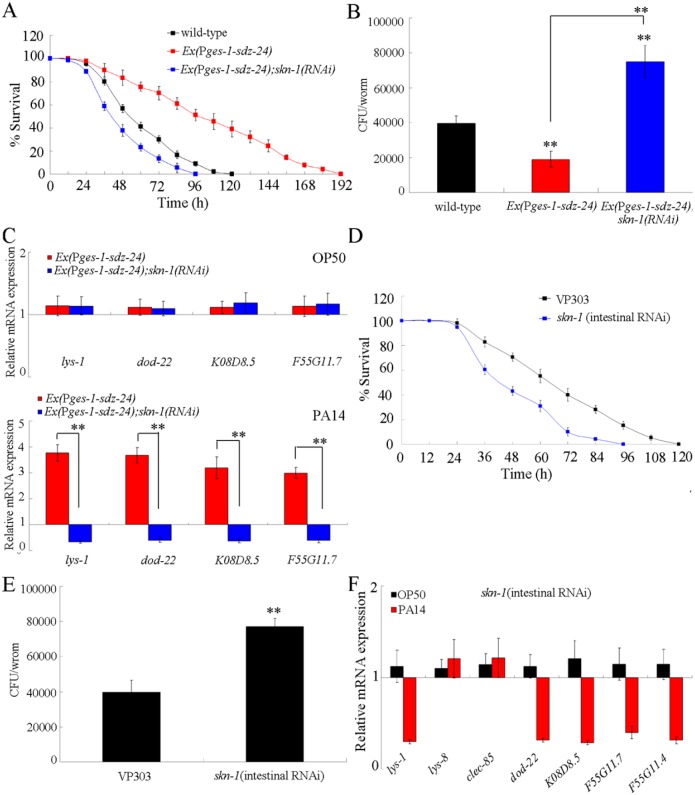
Genetic interaction between SDZ-24 and SKN-1 in the regulation of innate immune response to *P*. *aeruginosa* PA14 infection. (A) Genetic interaction between SDZ-24 and SKN-1 in the regulation of survival in *P*. *aeruginosa* PA14 infected nematodes. Statistical comparisons of the survival plots indicate that, after *P*. *aeruginosa* PA14 infection, the survival of *Ex(*P*ges-1-sdz-24);skn-1(RNAi)* was significantly different from that of *Ex(*P*ges-1-sdz-24)* (*P* < 0.0001). (B) Genetic interaction between SDZ-24 and SKN-1 in the regulation of CFU of *P*. *aeruginosa* PA14 in *P*. *aeruginosa* PA14 infected nematodes. (C) Genetic interaction between SDZ-24 and SKN-1 in the regulation of expression patterns of anti-microbial genes in *P*. *aeruginosa* PA14 infected nematodes. Normalized expression is presented relative to wild-type expression. (D) Effects of intestinal RNAi of *skn-1* gene on survival in *P*. *aeruginosa* PA14 infected nematodes. Statistical comparison of the survival plots indicate that, after *P*. *aeruginosa* PA14 infection, the survival of nematodes with intestinal RNAi of *skn-1* gene was significantly different from that of VP303 strain (*P* < 0.0001). (E) Effects of intestinal RNAi of *skn-1* gene on CFU of *P*. *aeruginosa* PA14 in the body of nematodes. (F) Effects of intestinal RNAi of *skn-1* gene on expression patterns of anti-microbial genes in *P*. *aeruginosa* PA14 infected nematodes. Normalized expression is presented relative to wild-type expression. Bars represent mean ± SD. **P < 0.01 *vs* wild-type (if not specially indicated).

Considering the fact that SDZ-24 is a SKN-1-dependent protein, we next investigated the genetic interaction between SDZ-24 and SKN-1 in the regulation of innate immune response to *P*. *aeruginosa* PA14 infection. After *P*. *aeruginosa* PA14 infection, RNAi knockdown of *skn-1* gene significantly reduced the survival in transgenic nematodes overexpressing intestinal *sdz-24* (Fig 9A), suggesting that RNAi knockdown of *skn-1* gene may suppress the resistant property of nematodes overexpressing intestinal *sdz-24* to *P*. *aeruginosa* PA14 infection. RNAi knockdown of *skn-1* gene further significantly increased the CFU of *P*. *aeruginosa* PA14 in the body of infected nematodes overexpressing intestinal *sdz-24* (Fig 9B). Moreover, RNAi knockdown of *skn-1* gene significantly inhibited the expression of antimicrobial genes (*lys-1*, *dod-22*, *K08D8*.*5*, and *F55G11*.*7*) in *P*. *aeruginosa* PA14 infected nematodes overexpressing intestinal *sdz-24* (Fig 9C). Therefore, the function of SDZ-24 in the regulation of innate immunity may be dependent on the SKN-1.

Previous study has demonstrated that SKN-1 is involved in the control of innate immunity [22]. We further found that, after *P*. *aeruginosa* PA14 infection, intestinal RNAi of *skn-1* gene significantly decreased the survival, enhanced the CFU of *P*. *aeruginosa* PA14 in the body, and suppressed the expression of antimicrobial genes (*lys-1*, *dod-22*, *K08D8*.*5*, *F55G11*.*7*, and *F55G11*.*4*) compared with VP303 (Fig 9D–9F), suggesting that *skn-1* gene can act in the intestine to regulate the innate immune response to *P*. *aeruginosa* PA14 infection.

### Genetic interaction of *sdz-24* with *daf-2* in the regulation of innate immune response to *P*. *aeruginosa* PA14 infection

**Fig 10:**
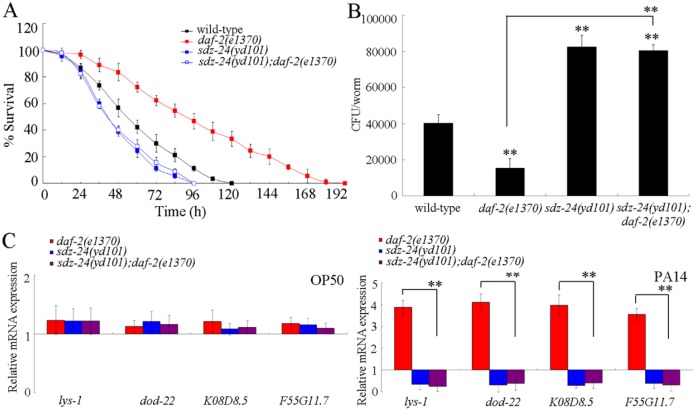
Genetic interaction between *sdz-24* and *daf-2* in the regulation of innate immune response to *P*. *aeruginosa* PA14 infection. (A) Genetic interaction between *sdz-24* and *daf-2* in the regulation of survival in *P*. *aeruginosa* PA14 infected nematodes. Statistical comparisons of the survival plots indicate that, after *P*. *aeruginosa* PA14 infection, the survival of *sdz-24(yd101);daf-2(e1370)* was not significantly different from that of *sdz-24(RNAi)* (*P* = 0.9218), and the survival of *sdz-24(yd101);daf-2(e1370)* was significantly different from that of *daf-2(e1370)* (*P* < 0.0001). (B) Genetic interaction between *sdz-24* and *daf-2* in the regulation of CFU of *P*. *aeruginosa* PA14 in the body of nematodes. (C) Genetic interaction between *sdz-24* and *daf-2* in the regulation of expression patterns of anti-microbial genes in *P*. *aeruginosa* PA14 infected nematodes. Normalized expression is presented relative to wild-type expression. Bars represent mean ± SD. **P < 0.01 *vs* wild-type (if not specially indicated).

The transcriptional factor of SKN-1/Nrf usually acts downstream of DAF-2, the insulin receptor in the insulin signaling pathway, to regulate the biological processes such as longevity [31]. We further asked whether SDZ-24 can function through the insulin signaling to regulate the innate immunity. We found that mutation of *sdz-24* gene significantly inhibited the resistant property of *daf-2(e1370)* mutant to *P*. *aeruginosa* PA14 infection (Fig 10A). Mutation of *sdz-24* gene also significantly increased the CFU of *P*. *aeruginosa* PA14 in the body of *P*. *aeruginosa* PA14 infected *daf-2(e1370)* mutant nematodes (Fig 10B). Moreover, mutation of *sdz-24* gene significantly suppressed the expression of antimicrobial genes (*lys-1*, *dod-22*, *K08D8*.*5*, and *F55G11*.*7*) in *P*. *aeruginosa* PA14 infected *daf-2(e1370)* mutant nematodes (Fig 10C). Therefore, SDZ-24 may act downstream of DAF-2 to regulate the innate immunity.

## Discussion

In *C*. *elegans*, the miRNA of *let-7* may have multiple biological functions by suppressing the functions of different targets. It has been shown that *let-7* is involved in the control of developmental timing [18–19]. In this study, we observed that *P*. *aeruginosa* PA14 infection could significantly decrease the expression of *let-7*::*GFP* (Fig 1). Meanwhile, the loss-of-function mutation of *let-7* could induce a resistant property to *P*. *aeruginosa* PA14 infection, suppress the *P*. *aeruginosa* PA14 colonization, and lead to an elevated innate immune response (Fig 1B and 1C) [20]. Therefore, *let-7* may encode a protection mechanism for nematodes against the toxic effects of *P*. *aeruginosa* PA14 infection. An emerging body of data has demonstrated the immune response induction as a source of cellular stress for nematodes [32–33]. Our data suggest the important physiological role of *let-7* in suppressing the immune response genes.

Previous study has implied that LIN-41 and HBL-1, two proteins required for the control of developmental timing, may act as the potential targets for *let-7* in the regulation of innate immunity [20]. In this study, we further identified the other potential targets for *let-7* in the regulation of innate immune response to *P*. *aeruginosa* PA14 infection. Based on the information from TargetScan prediction and dysregulated mRNA profiling induced by *P*. *aeruginosa* PA14 infection, we identified 7 candidate molecular targets for *let-7* in the regulation of innate immune response to *P*. *aeruginosa* PA14 infection (Fig 3A). Among these 7 candidate targets, SDZ-24 was identified as a direct target for *let-7* in the regulation of innate immune response to *P*. *aeruginosa* PA14 infection based on the assays of survival, CFU of *P*. *aeruginosa* PA14, expression pattern of antimicrobial genes, genetic interaction, and *in vivo sdz-24* 3’-UTR analysis (Figs 3B–3D, 4 and 7). These results imply that *let-7* could directly target both the developmental timing proteins and the non-developmental timing proteins, such as the SDZ-24, to regulate the innate immune response. In *C*. *elegans*, mutation of *sdz-24* did not affect the longevity (S8 Fig).

Nevertheless, we did not found that *P*. *aeruginosa* PA14 infection could significantly alter the expression levels of *lin-41* and *hbl-1* [25]. This implies that the altered expression of *let-7* may be not enough to affect the expression of its targets of LIN-41 and HBL-1 in nematodes infected with *P*. *aeruginosa* PA14. Or, besides the *let-7*, the function of LIN-41 and HBL-1 in the regulation of innate immunity may be also under the control of other miRNAs or signaling pathways. In this study, we also found NHR-43 did not act as a direct target for *let-7* in the regulation of innate immune response to *P*. *aeruginosa* PA14 infection (Fig 4A), although the *nhr-43* gene was involved in the control of innate immunity (Fig 3B–3D), implying that other still unidentified miRNAs may target the NHR-43 to regulate the innate immune response to *P*. *aeruginosa* PA14 infection.

The tissue-specific activity assay indicated that *let-7* could function in intestine to regulate the innate immune response to *P*. *aeruginosa* PA14 infection. The expression of *let-7* in the intestine could reduce the survival, increase the CFU of *P*. *aeruginosa* PA14, and decrease the expression of antimicrobial genes (*lys-1*, *dod-22*, *K08D8*.*5*, *F55G11*.*7*, and *F55G11*.*4*) in *let-7(mg279)* after *P*. *aeruginosa* PA14 infection (Fig 2). Overexpression of *let-7* in the intestine could reduce the survival, increase the CFU of *P*. *aeruginosa* PA14, and decrease the expression of antimicrobial genes (*lys-1*, *dod-22*, *K08D8*.*5*, *F55G11*.*7*, and *F55G11*.*4*) in *P*. *aeruginosa* PA14 infected wild-type nematodes (S3 Fig). In this study, we provide several lines of evidence to prove that SDZ-24 may act as a direct intestinal target for *let-7* in the regulation of innate immune response to *P*. *aeruginosa* PA14 infection. Firstly, after *P*. *aeruginosa* PA14 infection, loss-of-function of *let-7* significantly increased the expression of intestinal SDZ-24::GFP (S5 Fig). Secondly, intestinal RNAi of *sdz-24* gene could reduce the survival, increased the CFU of *P*. *aeruginosa* PA14, and suppressed the expression of antimicrobial genes (*lys-1*, *dod-22*, *K08D8*.*5*, and *F55G11*.*7*) after *P*. *aeruginosa* PA14 infection (Fig 5A–5C). Thirdly, after *P*. *aeruginosa* PA14 infection, overexpression of *sdz-24* gene in the intestine caused significant increase in the survival, decrease in the CFU of *P*. *aeruginosa* PA14, and enhancement of the expression of antimicrobial genes (*lys-1*, *dod-22*, *K08D8*.*5*, and *F55G11*.*7*) (Fig 5D–5F). More importantly, we found that the intestinal overexpression of *sdz-24* lacking 3’-UTR could effectively suppress the susceptible property of nematodes overexpressing intestinal *let-7* to *P*. *aeruginosa* PA14 infection (Fig 6A–6C). Different from this, we could observe the effects of intestinal expression of *let-7* on innate immunity in *P*. *aeruginosa* PA14 infected transgenic strain overexpressing *sdz-24* containing its 3’-UTR (Fig 6D–6F).

Moreover, our tissue-specific activity assay also indicated that *let-7* may further act in the neurons to regulate the innate immune response to *P*. *aeruginosa* PA14 infection. The expression of *let-7* in the neurons reduced the survival, increased the CFU of *P*. *aeruginosa* PA14, and decreased the expression of antimicrobial genes (*lys-1*, *dod-22*, *K08D8*.*5*, *F55G11*.*7*, and *F55G11*.*4*) in *let-7(mg279)* after *P*. *aeruginosa* PA14 infection (Fig 2). Additionally, overexpression of the *let-7* in the neurons also reduced the survival, increased the CFU of *P*. *aeruginosa* PA14, and decreased the expression of antimicrobial genes (*lys-1*, *dod-22*, *K08D8*.*5*, *F55G11*.*7*, and *F55G11*.*4*) in *P*. *aeruginosa* PA14 infect wild-type nematodes (S3 Fig). These results imply that *let-7* may further mediate certain neuronal signaling pathway(s) to regulate the innate immunity. However, so far, we still do not know the possible direct neuronal target for *let-7* in the regulation of innate immune response to *P*. *aeruginosa* PA14 infection.

For the intestinal SDZ-24-mediated signaling pathways, we found that the intestinal SDZ-24 may act upstream of SKN-1/Nrf to regulate the innate immunity, because RNAi knockdown the *skn-1* gene could significantly decrease the survival, increase the CFU of *P*. *aeruginosa* PA14, and inhibit the expression of antimicrobial genes (*lys-1*, *dod-22*, *K08D8*.*5*, and *F55G11*.*7*) in nematodes overexpressing intestinal *sdz-24* (Fig 9A–9C). It was reported that *P*. *aeruginosa* PA14 infection led to the intestinal SKN-1 accumulation [22]. In *C*. *elegans*, *mir-84* and *mir-241*, the other members in *let-7* family, could even regulate the innate immune response to *P*. *aeruginosa* PA14 infection by directly targeting SKN-1 [15]. It was reported that PMK-1 in the p38 MAPK signaling pathway acted upstream of SDZ-24 in the regulation of innate immunity [20]. In addition, SKN-1 could act downstream of p38 MAPK signaling pathway in the regulation of innate immunity [15]. These results imply the existence of signaling cascade of PMK-1-SDZ-24-SKN-1 in the intestine in the regulation of innate immunity. Meanwhile, after *P*. *aeruginosa* PA14 infection, intestinal RNAi knockdown of *skn-1* did not significantly affect the *sdz-24* expression (S9 Fig). In *C*. *elegans*, *sdz-24* encodes an ortholog of human RPA1, and may have nucleic acid binding activity based on the protein domain information [21, 34]. However, it is still unclear whether *sdz-24* can potentially have the nucleic acid binding activity with its targeted genes for the reason of lack of direct evidence.

In this study, we further found that intestinal SDZ-24 could act downstream of DAF-2 in the insulin signaling pathway to regulate the innate immunity, since RNAi knockdown the *sdz-24* gene reduced the survival, increased the CFU of *P*. *aeruginosa* PA14, and suppressed the expression of antimicrobial genes (*lys-1*, *dod-22*, *K08D8*.*5*, and *F55G11*.*7*) in *daf-2(e1370)* mutant nematodes (Fig 10). In *C*. *elegans*, both the *daf-2* gene and the *sdz-24* gene can act in the intestine to regulate the innate immunity (Fig 5) [8]. Therefore, in the intestine, the DAF-2-SDZ-24-SKN-1 signaling cascade may be also formed to be involved in the control of innate immune response to *P*. *aeruginosa* PA14 infection.

In conclusion, in this study, we examined the tissue-specific activity of *let-7* in the regulation of innate immune response to *P*. *aeruginosa* PA14 infection. We found that *let-7* could function in both the intestine and the neurons to regulate the innate immunity. During the control of innate immune response to *P*. *aeruginosa* PA14 infection, we identified the SDZ-24 protein as a direct target for *let-7* in the intestine. *let-7* could regulate the innate immune response to *P*. *aeruginosa* PA14 infection by suppressing both the expression and the function of intestinal SDZ-24. Moreover, we raised the SDZ-24-mediated signaling cascades potentially formed in the intestine for nematodes against the *P*. *aeruginosa* PA14 infection. Our results provide an important molecular basis for the intestinal *let-7* in the regulation of innate immunity. Our results will further highlight the crucial role of intestinal miRNAs for animals against the pathogen infection.

## Materials and Methods

### *C*. *elegans* strains

Nematodes strains used in the present study were wild-type N2, mutants of *let-7(mg279)*, *eat-2(ad465)*, *sdz-24(yd101)*, *sid-1(qt9)*, *tag-38(tm470)*, *nhr-43(tm1381)*, *mtl-1(tm1770)*, *nex-4(gk102)*, *daf-2(e1370)*, *Y95B8A*.*6(tm5312)*, *nhr-43(tm1381);let-7(mg279)*, *sdz-24(dy101);let-7(mg279)*, and *sdz-24(dy101);daf-2(e1370)*, and transgenic strains of *zaEx5*[*let-7*::*GFP*], *let-7(mg279)Ex(*P*ges-1-let-7)*, *let-7(mg279)Ex(*P*unc-14-let-7)*, *let-7(mg279)Ex(*P*myo-2-let-7)*, *let-7(mg279)Ex(*P*myo-3-let-7)*, *let-7(mg279)Ex(*P*dpy-7-let-7)*, *Is(*P*ges-1-let-7)*, *Is(*P*unc-14-let-7)*, *Ex(*P*sdz-24-sdz-24*::*GFP)*, *E*x(P*sdz-24-sdz-24*::*GFP);let-7(mg279)*, VP303/*kbIs7*[*nhx-2p*::*rde-1*], *Ex(*P*ges-1-sdz-24)*, *Ex(*P*ges-1-sdz-24);Is(*P*ges-1-let-7)*, *Ex(*P*ges-1-sdz-24-3’-UTR)*, *Ex(*P*ges-1-sdz-24-3’-UTR);Is(*P*ges-1-let-7)*, *daf-16(RNAi);Ex(*P*ges-1-sdz-24)***,** *Ex(*P*ges-1-sdz-24);dbl-1(RNAi)*, *Ex(*P*ges-1-sdz-24);pmk-1(RNAi)*, *Ex(*P*ges-1-sdz-24);sek-1(RNAi)*, *Ex(*P*ges-1-sdz-24)nsy-1(RNAi)*, and *Ex(*P*ges-1sdz-24);skn-1(RNAi)*. Nematodes were basically maintained on nematode growth medium (NGM) plates seeded with *E*. *coli* OP50 at 20°C as described [35].

### *P*. *aeruginosa* PA14 pathogenesis assay

Age synchronous populations of L4-larvae were prepared as described [36]. The L4-larvae were infected with *P*. *aeruginosa* PA14 as described [37]. *P*. *aeruginosa* PA14 was cultured in Luria broth, and seeded on killing plates containing a modified NGM (0.35% instead of 0.25% peptone). *P*. *aeruginosa* PA14 was incubated first at 37°C for 24-h and then at 25°C for 24-h. The *P*. *aeruginosa* PA14 infection was started by adding 60 animals to each plate at 25°C. Full-lawn PA14 killing plates were prepared for *P*. *aeruginosa* PA14 infection.

### Survival assay

Survival assay was performed basically as described [38]. During the *P*. *aeruginosa* PA14 infection, nematodes were scored for dead or live every 12-h. Nematodes would be scored as dead if no response was detected after prodding with a platinum wire. The hermaphrodite nematodes were transferred daily at 25°C for the first 5 days of adulthood. For survival assay, three replicates were performed. For the survival assay, the killing plates contained the modified NGM, as well as of fluoro-29-deoxyuridine (FUdR, 75 μ g/mL) to prevent the growth of progeny. Survival curve data were statistically analyzed using the log-rank test. The survival curves were considered to be significantly different from the control when the *p-*values were less than 0.01.

### *P*. *aeruginosa* CFU analysis

CFU of *P*. *aeruginosa* PA14 was analyzed as described [39]. After *P*. *aeruginosa* PA14 infection for 24-h, nematodes were transferred into 25 mM levamisole to paralyze nematodes and to stop the pharyngeal pumping. Nematodes were then placed on a NGM plate containing ampicillin (1 mg/mL) and gentamicin (1 mg/mL) for 15-min in order to eliminate the *P*. *aeruginosa* PA14 stuck to the body of animals. Nematodes were transferred onto a new NGM plate containing ampicillin (1 mg/mL) and gentamicin (1 mg/mL) for 30-min to further eliminate the external *P*. *aeruginosa* PA14. After that, the nematodes were lysed with a motorized pestle. The lysates were serially diluted with M9 buffer, and plated on Luria-Bertani plates containing rifampicin (100 μg/mL) for the selection of *P*. *aeruginosa* PA14. After incubation at 37°C overnight, colonies of *P*. *aeruginosa* PA14 were counted to determine the CFU per nematode. Ten nematodes were examined per treatment, and six replicates were performed.

### Quantitative real-time polymerase chain reaction (qRT-PCR)

The nematodes were infected with *P*. *aeruginosa* PA14 for 24-h. Total nematode RNA (~ 1 μg) was extracted using RNeasy Mini kit (Qiagen), and reverse-transcribed using a cDNA synthesis kit (Bio-Rad Laboratories). qRT-PCR was performed at the optimized annealing temperature of 58°C. The examined targeted genes were *lys-1*, *lys-8*, *clec-85*, *dod-22*, *K08D8*.*5*, *F55G11*.*7*, *F55G11*.*4*, *T24B8*.*5*, *F08G5*.*6*, and *F35E12*.*5*. Relative quantification of targeted genes in comparison to reference *tba-1* gene, encoding a tubulin, was determined. The final results were expressed as the relative expression ratio between the targeted gene and the reference *tba-1* gene. The designed primers for targeted genes and reference *tba-1* gene were shown in S1 Table. Six replicates were performed.

### Brood size assay

The brood size was analyzed as described [40]. To assay brood size, the number of offspring at all stages beyond the egg was counted. Ten nematodes were used for each reproduction assay, and three replicates were performed.

### Bioinformatics analysis for targeted gene prediction of *let-7*

The corresponding targeted genes for *let-7* were firstly predicted using the TargetScan software by searching for the presence of conserved sites that match the seed region of *let-7* (version 6.2, http://www.targetscan.org/worm_52/). This prediction results were confirmed by mirBase, PicTar, and miRanda assays. After that, we further screened whether the predicted targeted genes of *let-7* could also be dysregulated by *P*. *aeruginosa* PA14 infection [25].

### DNA constructs and germline transformation

To generate entry vector carrying promoter sequence, *ges-1* promoter used for the intestine-specific expression, *unc-14* promoter used for the neuron-specific expression, *myo-2* promoter used for the pharynx-specific expression, *myo-3* promoter used for the muscle-specific expression, or *dpy-7* promoter used for the hypodermis-specific expression was amplified by PCR from *C*. *elegans* genomic DNA. The *ges-1*, *unc-14*, *myo-2*, *myo-3*, or *dpy-7* promoter was inserted into pPD95_77 vector in the sense orientation. The *let-7*, or *sdz-24/K07E8*.*3*.*1* cDNA lacking 3’-UTR or containing 3’-UTR was amplified by PCR, verified by sequencing, and inserted into the corresponding entry vector behind the *ges-1*, *unc-14*, *myo-2*, *myo-3*, or *dpy-7* promoter. Transgenic nematodes were generated as described by coinjecting the testing DNA at a concentration of 10–40 μg/mL and a marker DNA (P*dop-1*::*rfp*) at a concentration of 60 μg/mL into the gonad of nematodes [41]. The designed primers for DNA construct generation were shown in S2 Table.

### Generation of *sdz-24* knockout strain using Cas9-triggered homologous recombination

The method was performed as described [26]. To generate deletion allele of *sdz-24*, we targeted the fragment corresponding to the first exon. To clone the sequence with the target sites into the sgRNA expression vector, we designed the primers of Guide F (5’-CCCTATTCCATCACTCACTC-3’), and Guide R (5’-TCCTGCTCACCGACTCGTT-3’). To target Cas9 to the genomic sequence of *sdz-24*, we inserted the desired targeting sequence into the Cas9-sgRNA construct (pDD162) using the Q5 site-directed mutagenesis kit (New England BioLabs) using the forward primer (5′-N19GTTTTAGAGCTAGAAATAGCAAGT-3′) and the reverse primer (5′-CAAGACATCTCGCAATAGG-3′). For this deletion construction, we injected 20 animals with a mixture containing 5 ng/ml Pmyo-3::mCherry (pCFJ104), and 50 ng/ml of each of the two sgRNAs using standard microinjection procedures as described [41]. From transgenic F1 animals expressing mCherry, a region surrounding the target site of *sdz-24* was PCR amplified using primers of *sdz-24* PL (5’- CACTTTCACAAATGCTCCGCCTA-3’), and *sdz-24* PR (5’- GGCCTGTGCGATTTGGATATCTT-3’) in order to confirm the knockout animals. The homozygous mutant lines were established by isolating single F2 nematodes and determining their genotype by PCR and sequence analysis.

### 3’-UTR reporters and microscopy

The 3’-UTR (wt) of *sdz-24* was PCR amplified from genomic DNA. A *sdz-24* 3’-UTR (mut) reporter was constructed by replacing putative *let-7* binding site with an oligonucleotide containing the exact complementary sequence of *let-7*. The synthesized *sdz-24* 3’-UTR (mut) sequence is ATAGAATTCTTTGCCGTGTGTAACCGAATGGCTCAATAAAGAGGGAAAGTGTCCAACATGCCGCAAGTTGCTTCTCAACCCCGGGATGATGGAGTATTGAATTAAATTTATAATATTTTTAGTGTCTCAAGTTTGTATTTTGAATGTATGAGAATATTTCGAAAAAATTATATCATGAATTACTTTCTTCTTATAACCTGGAACACAACAAAGGGGCCCTAT. The 3’ UTR reporter construct (P*ges-1*::*GFP-3’ UTR (sdz-24 wt)* or P*ges-1*::*GFP-3’ UTR (sdz-24 mut)*) and mCherry internal control (P*ges-1*::*mCherry-3’ UTR (tag-192)*) plasmid were coinjected into the gonad of nematodes as described [41]. The expression of GFP and mCherry was observed and analyzed under a fluorescence microscope (Olympus BX41, Olympus Corporation, Japan). The designed primers for related DNA construct generation were shown in S2 Table.

### RNAi assay

RNAi was performed by feeding nematodes with *E*. *coli* strain HT115 (DE3) expressing double-stranded RNA that is homologous to a target of *sdz-24*, *K01A2*.*10*, *daf-16*, *dbl-1*, *pmk-1*, *sek-1*, *nsy-1*, or *skn-1* gene as described [42]. *E*. *coli* HT115 (DE3) was first grown in LB broth containing ampicillin (100 μg/mL) at 37°C overnight, and then plated onto NGM plants containing ampicillin (100 μg/mL) and isopropyl 1-thio-β-D-galactopyranoside (IPTG, 5 mM). L2 larvae of certain strain were transferred onto RNAi plates for 2 days at 20°C until they developed into the gravid. The gravid adults were further transferred onto the fresh RNAi-expressing bacterial lawns to let them lay eggs for 2 h to obtain the second generation of RNAi population. The eggs were allowed to develop at 20°C to young adults for the subsequent survival, CFU of *P*. *aeruginosa* PA14, and gene expression pattern assays.

### Western blotting assay

The method was performed as described previously [43]. Nematode protein was extracted and electrophoresed on a 10% sodium dodecyl sulfate-polyacrylamide gel electrophoresis (SDS-PAGE) gel. The gel was transferred to a nitrocellulose membrane using a Bio-Rad semi-dry transfer apparatus. After pre-incubation with 5% nonfat milk in TBST buffer (10 mM Tris, pH 8.0, 150 mM NaCl and 0.5% Tween 20) for 30 min, the membrane was incubated with a primary antibody in TBST buffer with 5% nonfat milk for 12 h at 4°C. After washing with the TBST buffer, the membrane was further incubated with a horseradish peroxidase (HRP)-conjugated secondary antibody for 1.5 h. The membrane was then developed with ECL system (Thermo Scientific). Anti-phospho-p38 MAPK monoclonal antibody 28B10 (1:500) was from Cell Signaling, and anti-Actin monoclonal antibody MAB1501 (1:5000) was from EMD Millipore. The goat anti-mouse IgG antibody (H&L) [HRP] (1:10 000) was from GenScript. Three replicates were performed.

### Statistical analysis

All data in this article were expressed as means ± standard deviation (SD). Graphs were generated using Microsoft Excel (Microsoft Corp., Redmond, WA). Statistical analysis was performed using SPSS 12.0 (SPSS Inc., Chicago, USA). Differences between groups were determined using analysis of variance (ANOVA). Probability levels of 0.05 and 0.01 were considered statistically significant.

## Supporting Information

S1 Fig Accumulation of PA14::GFP in the lumen of pharynx in wild-type and mutant nematodes. (DOC)

S2 Fig Brood size in wild-type and *let-7* mutant nematodes. (DOC)

S3 Fig Effects of *let-7* overexpression in the intestine or the neurons on innate immunity. (DOC)

S4 Fig Generation of *sdz-24* deletion by CRISPR/Cas9. (A) Gene predictions with sgRNA target sites.
(DOC)

S5 Fig *let-7* mutation altered the expression of SDZ-24::GFP. (DOC)

S6 Fig Effects of intestinal RNAi of *sdz-24* gene on innate immune response to *P*. *aeruginosa* PA14 infection. (DOC)

S7 Fig Effect of *sdz-24* mutation on expression of immune effectors of p38 MAPK signaling pathway and phosphorylated PMK-1 in *P*. *aeruginosa* PA14 infected nematodes. (DOC)

S8 Fig Mutation of *sdz-24* did not affect the longevity. (DOC)

S9 Fig Effect of intestinal RNAi knockdown of *skn-1* on transcriptional expression of *sdz-24* after *P*. *aeruginosa* PA14 infection. (DOC)

S1 Table Primers used for quantitative real-time polymerase chain reaction (PCR). (DOC)

S2 Table Primers for DNA construct generation. (DOC)
