# Supplementary material for: Molecular Control of Innate Immune Response to Pseudomonas aeruginosa Infection by Intestinal let-7 in Caenorhabditis elegans
Source: PLoS Pathog. 2017 Jan 17;13(1):e1006152. doi: 10.1371/journal.ppat.1006152 (PMC5271417; doi:10.1371/journal.ppat.1006152)
Supplement: S1 Fig — (DOC) [file ppat.1006152.s001.doc]

**
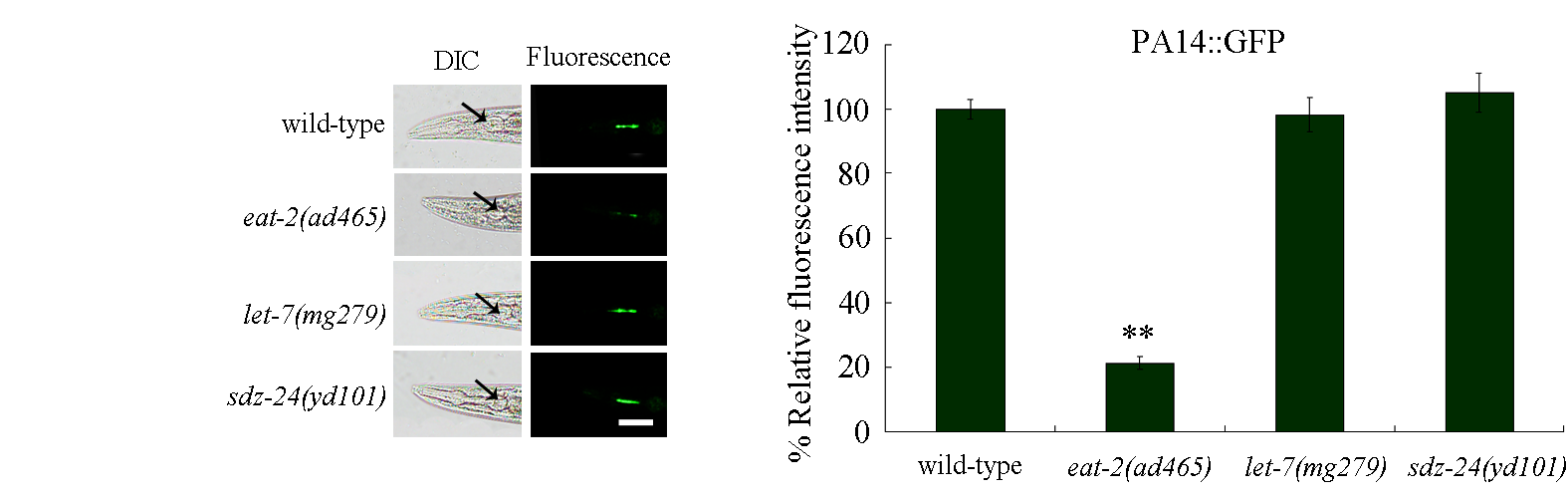
**

**Figure S1.** **Accumulation of PA14::GFP in the lumen of pharynx in wild-type and mutant nematodes.**  Arrowheads indicate the pharynx. Nematodes were infected with *P. aeruginosa* PA14 for 24-h. In the feeding mutant of *eat-2(ad465)*, we observed the significant decrease in the accumulation of PA14::GFP in the lumen of pharynx. Thirty animals were examined. Bars represent mean ± SD. ^**^*P* < 0.01 *vs* wild-type. Scale bar, 50 μm.
