# Supplementary figures and images for: Molecular Control of Innate Immune Response to Pseudomonas aeruginosa Infection by Intestinal let-7 in Caenorhabditis elegans
Source: PLoS Pathog. 2017 Jan 17;13(1):e1006152. doi: 10.1371/journal.ppat.1006152 (PMC5271417; doi:10.1371/journal.ppat.1006152)

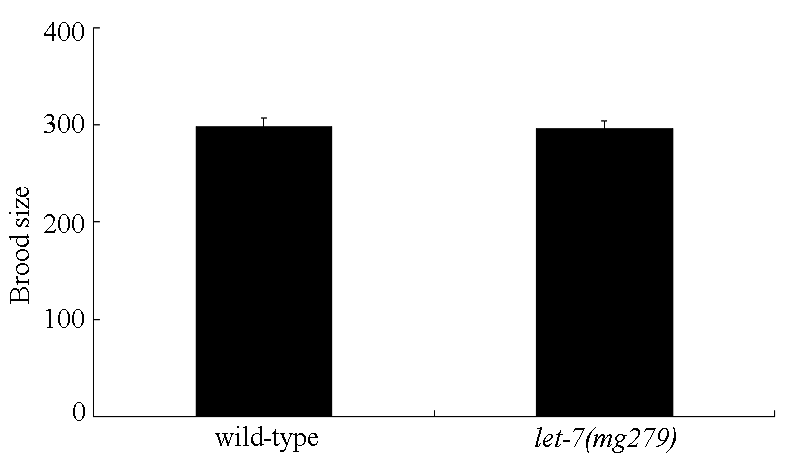


**Figure S2.** **Brood size in wild-type and *let-7* mutant nematodes.**  Bars represent mean ± SD.

Supplement: S2 Fig — (DOC) [file ppat.1006152.s002.doc]

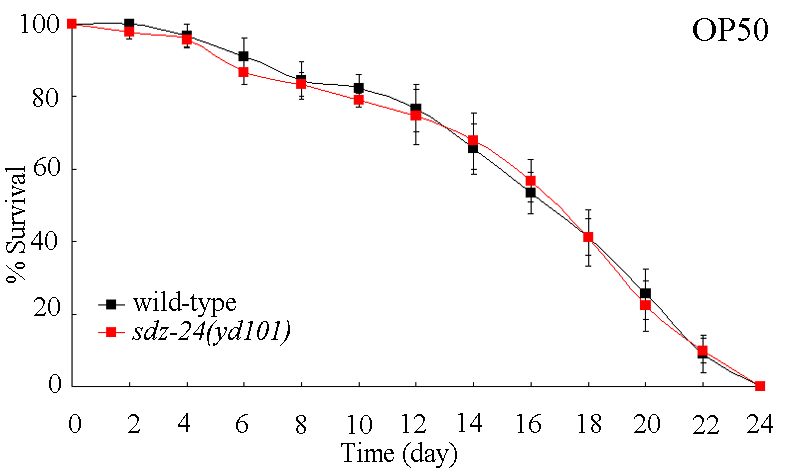


**Figure S8. Mutation of *sdz-24* did not affect the longevity.** Bars represent mean ± SD.

Supplement: S8 Fig — (DOC) [file ppat.1006152.s008.doc]
