# Supplementary material for: Molecular Control of Innate Immune Response to Pseudomonas aeruginosa Infection by Intestinal let-7 in Caenorhabditis elegans
Source: PLoS Pathog. 2017 Jan 17;13(1):e1006152. doi: 10.1371/journal.ppat.1006152 (PMC5271417; doi:10.1371/journal.ppat.1006152)
Supplement: S3 Fig — (DOC) [file ppat.1006152.s003.doc]

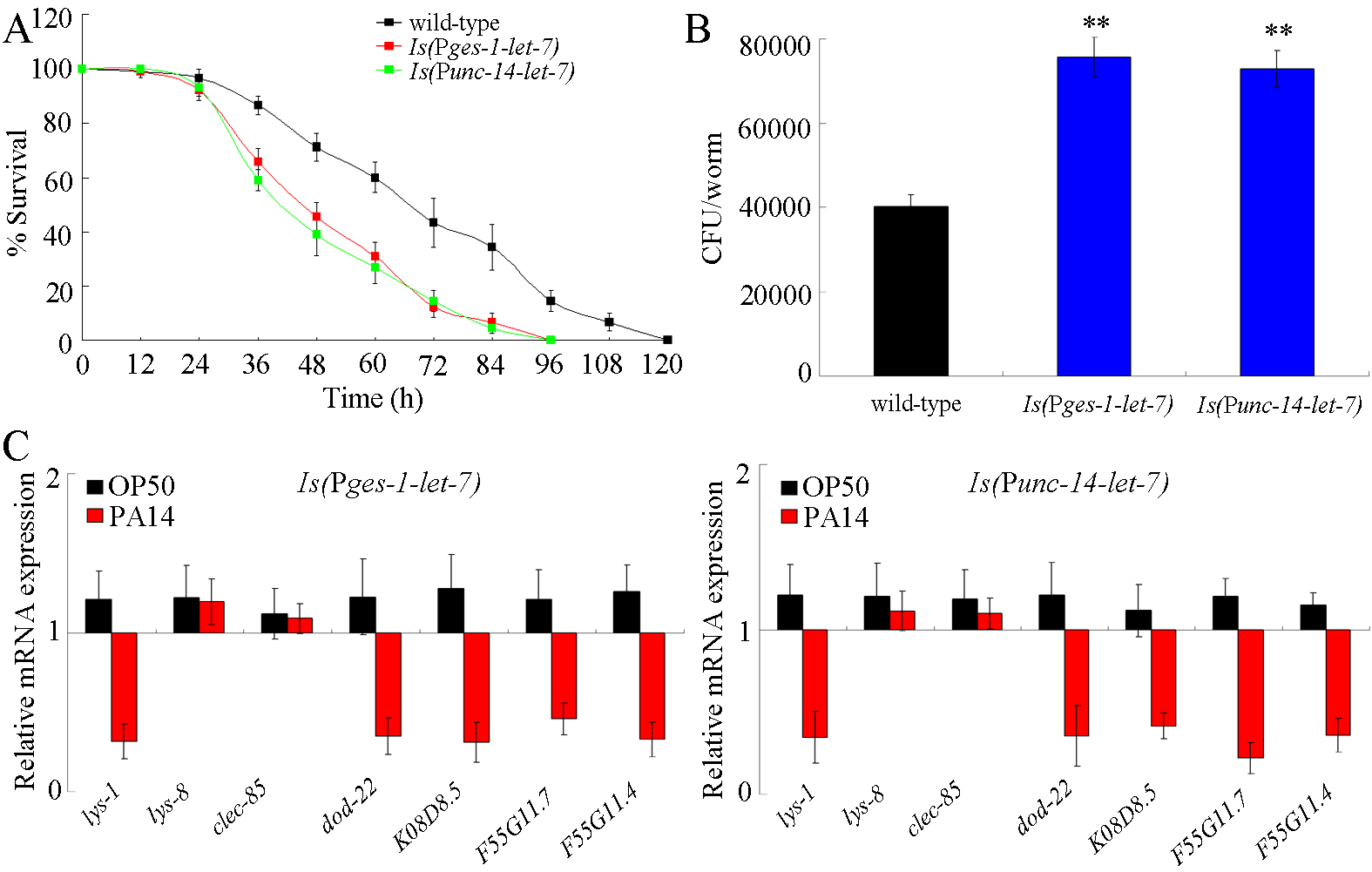


**Figure S3. Effects of *let-7* overexpression in the intestine or the neurons on innate immunity.** (A) Effects of *let-7* overexpression in the intestine or the neurons on survival in *P. aeruginosa* PA14 infected nematodes. Statistical comparisons of the survival plots indicate that, after *P. aeruginosa* PA14 infection, the survival of transgenic strain of *Is(*P*ges-1-let-7)* or *Is(*P*unc-14-let-7)* was significantly different from that of wild-type (*P* < 0.0001). (B) Effects of *let-7* overexpression in the intestine or the neurons on CFU of *P. aeruginosa* PA14 in the body of nematodes. (C) Effects of *let-7* overexpression in the intestine or the neurons on expression patterns of anti-microbial genes in *P. aeruginosa* PA14 infected nematodes. Normalized expression is presented relative to wild-type expression. Bars represent mean ± SD. ^**^*P* < 0.01 *vs* wild-type.
