# Supplementary material for: Molecular Control of Innate Immune Response to Pseudomonas aeruginosa Infection by Intestinal let-7 in Caenorhabditis elegans
Source: PLoS Pathog. 2017 Jan 17;13(1):e1006152. doi: 10.1371/journal.ppat.1006152 (PMC5271417; doi:10.1371/journal.ppat.1006152)
Supplement: S4 Fig — (A) Gene predictions with sgRNA target sites. (DOC) [file ppat.1006152.s004.doc]

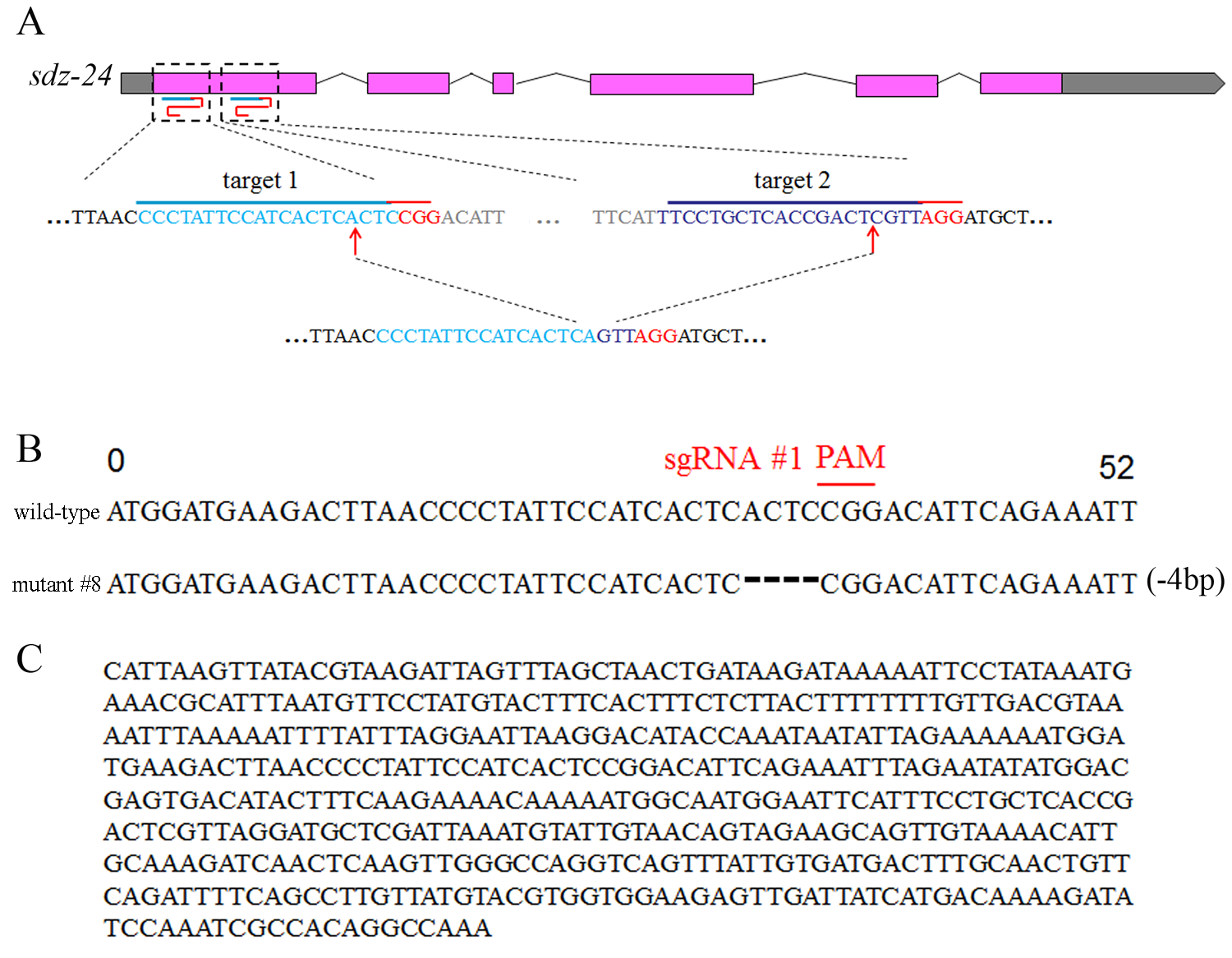


**Figure S4. Generation of *sdz-24* deletion by CRISPR/Cas9. (A) Gene predictions with sgRNA target sites.** Sequences of the target sites and expected deletion are shown. (B) Sequence alignments of wild-type and mutant animals. The PAM sequence is labeled in red and overlined. The number of deleted (4) bases is shown on the right. Numbers on the top of sequences indicate positions relative to the transcription start site. (C) The sequencing data for mutant #8.
