# Supplementary material for: Molecular Control of Innate Immune Response to Pseudomonas aeruginosa Infection by Intestinal let-7 in Caenorhabditis elegans
Source: PLoS Pathog. 2017 Jan 17;13(1):e1006152. doi: 10.1371/journal.ppat.1006152 (PMC5271417; doi:10.1371/journal.ppat.1006152)
Supplement: S5 Fig — (DOC) [file ppat.1006152.s005.doc]

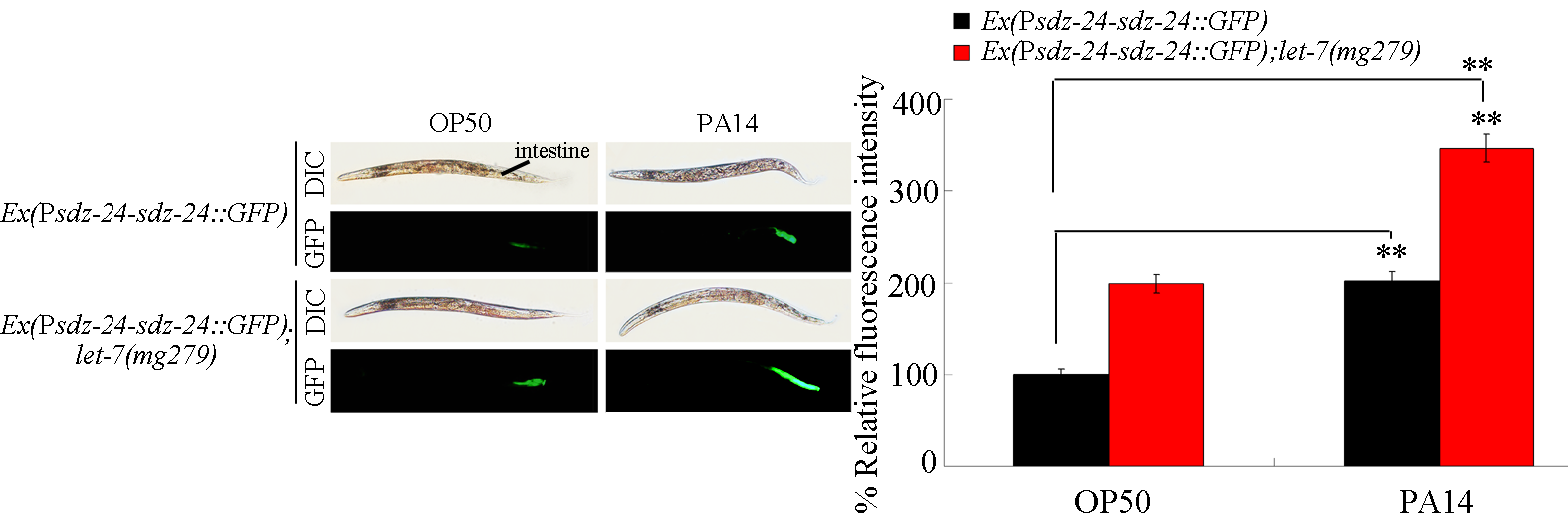


**Figure S5. *let-7* mutation altered the expression of SDZ-24::GFP.** Nematodes were infected with *P. aeruginosa* PA14 for 24-h. Bars represent mean ± SD. ^**^*P* < 0.01 *vs* OP50 (if not specially indicated).
