# Supplementary material for: Molecular Control of Innate Immune Response to Pseudomonas aeruginosa Infection by Intestinal let-7 in Caenorhabditis elegans
Source: PLoS Pathog. 2017 Jan 17;13(1):e1006152. doi: 10.1371/journal.ppat.1006152 (PMC5271417; doi:10.1371/journal.ppat.1006152)
Supplement: S6 Fig — (DOC) [file ppat.1006152.s006.doc]

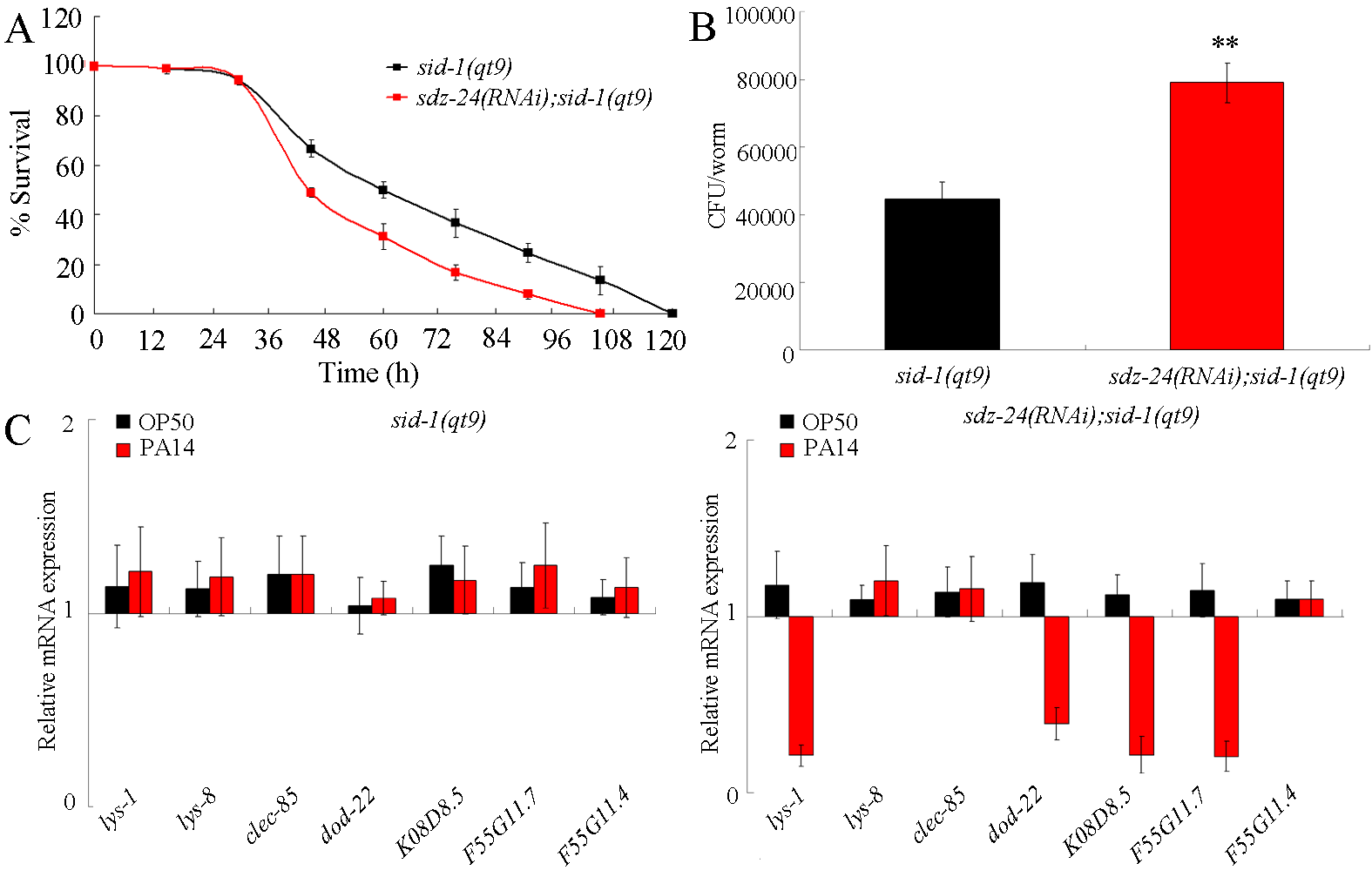


**Figure S6. Effects of intestinal RNAi of *sdz-24* gene on innate immune response to *P. aeruginosa* PA14 infection.** (A) Effects of intestinal RNAi of *sdz-24* gene on survival in *P. aeruginosa* PA14 infected nematodes. Statistical comparison of the survival plots indicate that, after *P. aeruginosa* PA14 infection, the survival of nematodes with intestinal RNAi of *sdz-24* gene was significantly different from that of *sid-1(qt9)* (*P* < 0.0001). Bars represent mean ± SD. (B) Effects of intestinal RNAi of *sdz-24* gene on CFU of *P. aeruginosa* PA14 in the body of nematodes. Bars represent mean ± SD. ^**^*P* < 0.01 *vs sid-1(qt9)*. (C) Effects of intestinal RNAi of *sdz-24* gene on expression patterns of anti-microbial genes in *P. aeruginosa* PA14 infected nematodes. Normalized expression is presented relative to wild-type expression for *sid-1(qt9)*. Normalized expression is presented relative to *sid-1(qt9)* for *sdz-24(intestinal RNAi)*. Bars represent mean ± SD.
