# Supplementary material for: Molecular Control of Innate Immune Response to Pseudomonas aeruginosa Infection by Intestinal let-7 in Caenorhabditis elegans
Source: PLoS Pathog. 2017 Jan 17;13(1):e1006152. doi: 10.1371/journal.ppat.1006152 (PMC5271417; doi:10.1371/journal.ppat.1006152)
Supplement: S7 Fig — (DOC) [file ppat.1006152.s007.doc]

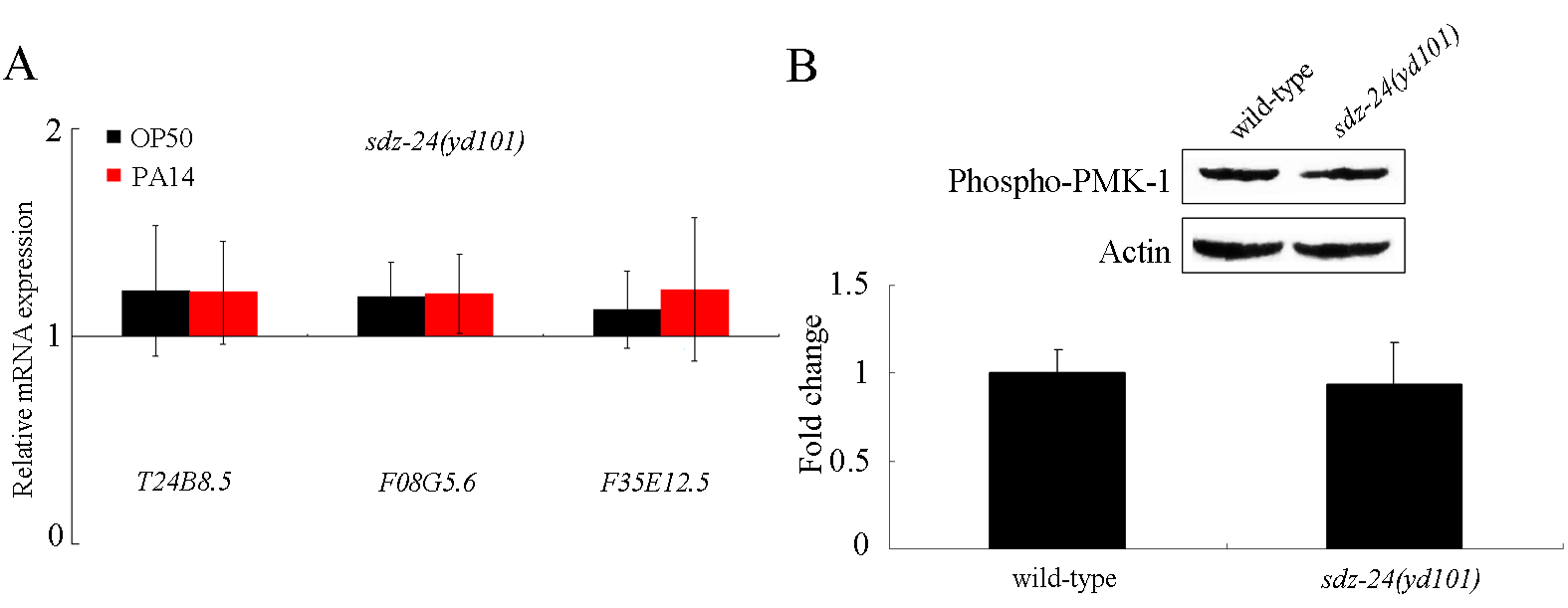


**Figure S7. Effect of *sdz-24* mutation on expression of immune effectors of p38 MAPK signaling pathway and phosphorylated PMK-1 in *P. aeruginosa* PA14 infected nematodes.** (A) Effect of *sdz-24* mutation on expression of immune effectors of p38 MAPK signaling pathway in *P. aeruginosa* PA14 infected nematodes. Normalized expression is presented relative to wild-type expression. (B) Western blotting analysis on the effect of *sdz-24* mutation on expression of phosphorylated PMK-1 in *P. aeruginosa* PA14 infected nematodes. Actin protein was used as the loading control. Bars represent mean ± SD.
