# Supplementary material for: Molecular Control of Innate Immune Response to Pseudomonas aeruginosa Infection by Intestinal let-7 in Caenorhabditis elegans
Source: PLoS Pathog. 2017 Jan 17;13(1):e1006152. doi: 10.1371/journal.ppat.1006152 (PMC5271417; doi:10.1371/journal.ppat.1006152)
Supplement: S9 Fig — (DOC) [file ppat.1006152.s009.doc]

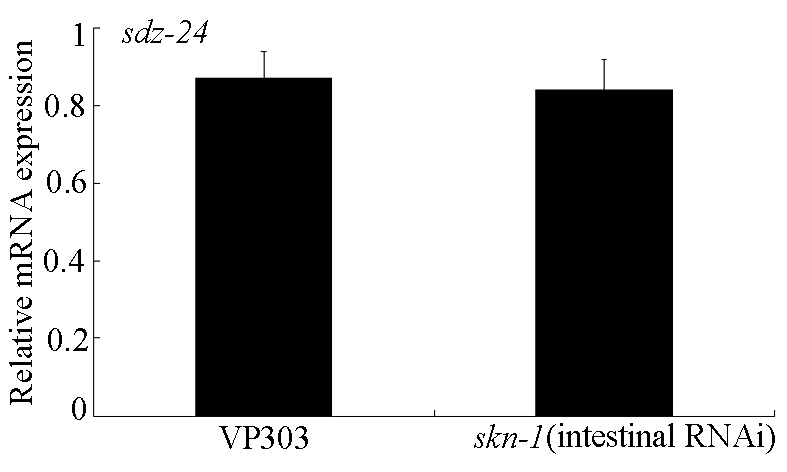


**Figure S9. Effect of intestinal RNAi knockdown of *skn-1* on transcriptional expression of *sdz-24* after *P. aeruginosa* PA14 infection.** Nematodes were infected with *P. aeruginosa* PA14 for 24-h. Bars represent mean ± SD.
