# Supplementary material for: Molecular Control of Innate Immune Response to Pseudomonas aeruginosa Infection by Intestinal let-7 in Caenorhabditis elegans
Source: PLoS Pathog. 2017 Jan 17;13(1):e1006152. doi: 10.1371/journal.ppat.1006152 (PMC5271417; doi:10.1371/journal.ppat.1006152)
Supplement: S1 Table — (DOC) [file ppat.1006152.s010.doc]

**Table S1. Primers used for** quantitative real-time polymerase chain reaction (PCR)

| Gene | Forward primer (5’-3’) | Reverse primer (5’-3’) |
| --- | --- | --- |
| *tba-1* | TCAACACTGCCATCGCCGCC | TCCAAGCGAGACCAGGCTTCAG |
| *lys-1* | TTCGGATCTTTCAAGAAG | TGGGATTCCAACAACGTA |
| *lys-8* | TCAGTCTCCGTCAAGGTC | GAAGCTGGCTCAATGAAA |
| *clec-85* | GGTTTTGGCTGTAGCACG | GGTTTTGGCTGTAGCACG |
| *dod-22* | CCAGGATACAGAATACGT | CCAGAGATGACTTCAGTT |
| *K08D8.5* | TTACGATGGTGATTCCGT | GCTTGTTGCCAGTTGAGA |
| *F55G11.7* | CACCCTCAGGCCAACTCA | CTGTGACTGTAGCGTCAC |
| *F55G11.4* | GGATCCGTGTATTTGGCT | GTGAAGACATATGTGCTC |
| *T24B8.5* | AAGACCATCATGCCCTTCAC | CCACAGATTTGGCAGGTTTT |
| *F08G5.6* | CACAATGATTTCAATGCGAGA | GTTTCGACCGAGAAATCGAG |
| *F35E12.5* | ACACAATCATTTGCGATGGA | GGTAGTCATTGGAGCCGAAA |
| *sdz-24* | TCACCGACTCGTTAGG | CTGTGCGATTTGGATA |

**Table S2. Primers for DNA construct generation**

| Gene | Forward primer (5’-3’) | Reverse primer (5’-3’) |
| --- | --- | --- |
| P*ges-1* | ATATCTAGAAGCCACTCAGCCACTTCA | ATAGGATCCCATCTGAATTCAAAGATA |
| P*unc-14* | ATAAAGCTTCCATCAGTTAAAACCTGT | ATAGGATCCCATTTTGGTGGAAGAATT |
| P*myo-2* | CCCAAGCTTGGTGGTGGACAGTAACTGTCTGT | CGCTCTAGACATTTCTGTGGTCTGACGATCGA |
| P*myo-3* | CTCAAGCTTCACTTCCGGCGCCCTGAA | TAGGGATCCCATTTCTAGATGGATCTA |
| P*dpy-7* | TACAAGCTTCTATGTGCAATGTCACGTGGA | CGCGGATCCCTGGAACAAAATGTAAGAATA |
| *let-7* | TATCCCGGGATGAGTAGCCCACCTAGC | CGGGGTACCACATTACCGATACAACAG |
| P*sdz-24* | ATAAAGCTTTAGCTCTAATAGGCACCC | CGCTCTAGACATTTTTTCTAATATTAT |
| *sdz-24* lacking 3’ UTR | ATAGGATCCATGGATGAAGACTTAACC | CGCGGTACCTTAAAGTTTCATCTTATT |
| *sdz-24* containing 3’ UTR | ATACCCGGGATGGATGAAGACTTAACC | ATAGGTACCCATGATATAATTTTTTCG |
| *sdz-24* 3’ UTR (wt) | ATAGAATTCTTTGCCGTGTGTAACCGA | ATAGGGCCCCTTTGTTGTGTTCCAGGT |
| *tag-192* 3’ UTR | ATAGAATTCTTCCTTACTAGAAATGTT | ATAGGGCCCATTACCCATTACATGAGA |
