# Supplementary material for: Molecular Control of Innate Immune Response to Pseudomonas aeruginosa Infection by Intestinal let-7 in Caenorhabditis elegans
Source: PLoS Pathog. 2017 Jan 17;13(1):e1006152. doi: 10.1371/journal.ppat.1006152 (PMC5271417; doi:10.1371/journal.ppat.1006152)
Supplement: S2 Table — (DOC) [file ppat.1006152.s011.doc]

**Table S2. Primers for DNA construct generation**

| Gene | Forward primer (5’-3’) | Reverse primer (5’-3’) |
| --- | --- | --- |
| P*ges-1* | ATATCTAGAAGCCACTCAGCCACTTCA | ATAGGATCCCATCTGAATTCAAAGATA |
| P*unc-14* | ATAAAGCTTCCATCAGTTAAAACCTGT | ATAGGATCCCATTTTGGTGGAAGAATT |
| P*myo-2* | CCCAAGCTTGGTGGTGGACAGTAACTGTCTGT | CGCTCTAGACATTTCTGTGGTCTGACGATCGA |
| P*myo-3* | CTCAAGCTTCACTTCCGGCGCCCTGAA | TAGGGATCCCATTTCTAGATGGATCTA |
| P*dpy-7* | TACAAGCTTCTATGTGCAATGTCACGTGGA | CGCGGATCCCTGGAACAAAATGTAAGAATA |
| *let-7* | TATCCCGGGATGAGTAGCCCACCTAGC | CGGGGTACCACATTACCGATACAACAG |
| P*sdz-24* | ATAAAGCTTTAGCTCTAATAGGCACCC | CGCTCTAGACATTTTTTCTAATATTAT |
| *sdz-24* lacking 3’ UTR | ATAGGATCCATGGATGAAGACTTAACC | CGCGGTACCTTAAAGTTTCATCTTATT |
| *sdz-24* containing 3’ UTR | ATACCCGGGATGGATGAAGACTTAACC | ATAGGTACCCATGATATAATTTTTTCG |
| *sdz-24* 3’ UTR (wt) | ATAGAATTCTTTGCCGTGTGTAACCGA | ATAGGGCCCCTTTGTTGTGTTCCAGGT |
| *tag-192* 3’ UTR | ATAGAATTCTTCCTTACTAGAAATGTT | ATAGGGCCCATTACCCATTACATGAGA |
